# Use of long-acting reversible contraception among adolescents and young women in Kenya

**DOI:** 10.1371/journal.pone.0241506

**Published:** 2020-11-10

**Authors:** Wambui Kungu, Anne Khasakhala, Alfred Agwanda

**Affiliations:** Population Studies and Research Institute, University of Nairobi, Nairobi, Kenya; University of Salamanca, SPAIN

## Abstract

The Kenya Demographic and Health Survey (KDHS 2014) revealed changing patterns in the contraceptive use of young women aged 15–24, shifting from injectable methods to implants. Long-acting reversible contraception (LARC) is user friendly, long-term, and more effective than other modern methods. It could be a game-changer in dealing with unintended pregnancies and herald a new chapter in the reproductive health and rights of young women. This study determined the factors associated with LARC use among adolescent girls and young women to expand the evidence of its potential as the most effective method of reducing unwanted pregnancies among the cohort. This study analysed secondary data from KDHS 2014 using binary logistic regression. The findings showed a rise in LARC use (18%), with identified predictors of reduced odds being aged 15–19 [OR = 0.735, 95% CI = 0.549–0.984], residence (rural) [OR = 0.674, CI = 0.525–0.865], religion (Protestant/other Christian) [OR = 0.377, CI = 0.168–0.842], married, [OR = 0.746, CI = 0.592–0.940], and region (high contraception) [OR = 0.773, CI = 0.626–0.955], while the number of living children showed increased odds for 1–2 children [OR = 17.624, CI = 9.482–32.756] and 3+ children [OR = 23.531, CI = 11.751–47.119]. This study established the rising popularity of LARC and identified factors that can be addressed to promote it. Its increased uptake could help Kenya achieve the International Conference on Population and Development 25’s first and second commitments on teenage pregnancies and maternal and new-born health, thus promoting the health, wellbeing, educational goals, and rights of this critical cohort. This study can guide the accelerated efforts needed in Kenya’s march towards the five zeros of unmet need for contraception, teenage pregnancies, unsafe abortions, preventable maternal deaths, and preventable neonatal/infant deaths.

## Introduction

In Kenya, teenage pregnancies account for 18% of the school dropouts and deaths of adolescents aged between 16–18 years old. These pregnancies are mostly unintended and are associated with additional negative health outcomes such as sexually transmitted infections/HIV/AIDS, unsafe abortions, miscarriages, and complications during birth that can leave victims with lifelong health challenges [1]. Adolescent girls aged below 19 constitute 20% of the patients who undergo post-abortion services in Kenyan health facilities, as well as 50% of those admitted with severe complications [2].

There are approximately 5 million adolescent girls and young women aged 15–24 in Kenya, which is more than 10% of the latest population figure of 47.6 million [3]. Therefore, it is imperative to acknowledge, understand, and respond to their reproductive health needs to minimise unintended pregnancies that compromise quality health services and sometimes lead to unsafe abortions and deaths from pregnancy and childbirth complications, especially for adolescents whose bodies have not matured [4].

Reducing teenage pregnancies from 18% to 12% by 2020 and 10% by 2025 was a Family Planning 2020 (FP2020) commitment for Kenya in 2017, but the targets have changed following commitments made at the International Conference on Population and Development 25 (ICPD25), Nairobi Summit held in 2019. The first ICPD25 commitment is to eliminate adolescent pregnancies in an effort to achieve universal health coverage for quality reproductive health services for adolescents and youth by 2030, and the second is to eliminate preventable maternal and new-born deaths [5]. The issue is also addressed in the Sustainable Development Goal number 3, target 3.2.

In the last two decades, Kenya has made remarkable progress in its uptake of contraception and has reduced the unmet need for contraception among all women of reproductive age. However, the pace has not been as fast for adolescents and young women, and inequalities are evident [6]. Concerted efforts can help reduce this gap [7] and increase the universal health coverages’ family planning index from 70% to almost 100% [8].

Long-acting reversible contraception (LARC), which refers to implants and intra-uterine devices (IUDs), was not previously encouraged among adolescents and young women, and only accounted for 2% of use in 2008/09. However, by 2014, the uptake of implants had risen almost tenfold, and had replaced the contraceptive pill and condoms to become the second most popular contraceptive (after injections) for this segment of women in Kenya [9, 10]. LARC was recommended by the World Health Organization as being safe and suitable for adolescent girls and young women, including nulliparous girls [11], and was among the factors that contributed to its increased usage.

The Health Act (2017) reinstated the right of every Kenyan woman to safe, effective, acceptable, and affordable contraception services [12], while many recent family planning policy documents have emphasised the prioritisation of LARC because it is longer acting, safer, more convenient, and highly effective [13]. Moreover, FP2020 issued a global statement promoting the expansion of method mix for adolescent girls and young women by including LARC [14].

The 2014 Kenya Demographic and Health Survey (KDHS) reported increased sexual activity among adolescent girls and young women without the use of effective contraception, and that 90% of sexually active adolescents and young women may end up with unintended pregnancies within a year of having unprotected sex [15]. Indeed, KDHS 2014 reported that 15% to 40% of adolescents aged 17–19, who should ideally be in school, have started to bear children. This results in increased adverse social consequences, as teenage mothers are more likely to drop out of school and lose out on education, career advancement, and social status [6]. LARC can help these young women avoid or delay pregnancies and reduce the incidence of maternal mortality [4], and thus aid the progress towards achieving the ICPD25 commitments 1 and 2 for Kenya.

LARC is the most effective contraceptive method (99% effective) and is 100 times more successful than the injection or contraceptive pill combined if used correctly in the first year, and thus reduces the risk of unwanted pregnancy by half [13]. Evidence has shown that more than 60% of adolescents and young women would readily utilise it if they were given comprehensive counselling by health providers [13]. A recent study in the USA provided evidence that the increased uptake of LARC has drastically reduced unintended pregnancies and abortions [16].

LARC regulates fertility for three to five years and can reduce rapid repeat pregnancies, which adolescents are at higher risk of, and can also deal with the challenge of the incorrect and inconsistent use of contraceptives, which is the major cause of unintended pregnancies [17]. LARC can reduce discontinuation, which is common among adolescent girls and young women, because it is long-term and has no challenges in its adherence. It also has high user satisfaction, is not user-dependent, and comes with some non-contraceptive benefits such as reducing menstrual pain/endometriosis and anaemia by raising haemoglobin levels [18]. Providing more information on LARC may promote its use among sexually active unmarried young women and consequently reduce unintended pregnancies [19].

Despite the numerous benefits of LARC, young women aged under 25 mostly use short-acting contraception such as pills, condoms, and injections, because service providers offer these methods under what can be called provider bias [20]. Provider bias refers to providers that withhold contraceptive information or services regarding some methods—which goes against ethical guidelines and is for reasons unrelated to the medical condition of the client—and thus creates a barrier on informed choice and wider method mix. It is based on concerns about the suitability of a method due to the age, marital status, and parity of a client, and is more pronounced for adolescents and young women [21]. Provider bias should be eliminated to reduce the unmet need for contraception and to meet the reproductive goals of contraceptive clients. Health workers should provide all available information and counsel to allow adolescent girls the freedom to choose LARC if they wish to enhance client satisfaction and a continuation of the methods [22].

Adolescent girls also harbour some misconceptions about LARC regarding their short- and long-term side effects, which they believe could negatively affect their fertility in the future. One of the misconceptions associated with IUDs is the increased risk of pelvic inflammatory disease in nulliparous users, but no evidence supports this claim [23]. Other concerns include fear of side effects such as weight gain and changes in the menstrual cycle, as well as a delayed return to fertility upon removal of the methods, more so for implants [24]. These misconceptions can be addressed with the provision of comprehensive information and counselling on LARC.

Much literature exists on modern contraceptive use, but there is a dearth of the same for adolescent girls and young women in Kenya, especially for LARC methods, which have only recently become popular among the group aged 15–24. Performance Monitoring and Accountability 2020 provides periodic briefs on modern contraceptive prevalence in adolescent girls and young women, but the factors that underlie the figures are not well understood [25, 26]. Studies of the determinants of LARC use among the sub-population are thus needed to both establish the barriers that potential users face and to inform the design of targeted programmes that can improve the accessibility, availability, and acceptability of the methods among these young women [27].

Kenya’s current Family Planning Policy, which was articulated in the Costed Implementation Plan (CIP) 2017–2020, advocates for the use of modern methods and LARC because of their efficacy, convenience, ease of use, continuation rates, and long-term nature. The policy recognises that sexually active young women aged 15–24 use less effective methods of contraception because they have little information on LARC. The CIP has the target of increasing the use of modern methods among this age group by 10% for unmarried women (from 49.3%) and married women (from 36.8%) [28]. In light of this, this study seeks to answer three critical questions regarding assessment and policy concerns: (1) What are the proportions of adolescent girls and young women using LARC? (2) what factors influence the choice of LARC methods? and (3) are there differences between adolescent girls and young women from lower and higher socio-economic stratums? The main objective was to determine the factors associated with LARC use among adolescents and young women aged 15–24 to expand the evidence for LARC’s potential as the most effective method of reducing unwanted pregnancies among the vulnerable cohort.

## Methodology

### Ethical statement

Specific ethical approval is not required for secondary analysis of DHS data but permission to use the data was obtained from ICF Macro. The secondary analysis was done under the original consent provided by participants.

### Data sources

This study used national and secondary data from the KDHS 2014. The KDHS is a national cross-sectional survey that monitors population and health indicators such as household characteristics, fertility, and maternal and child health, and is conducted every five years by the Government of Kenya. In this study, information was collected using three questionnaires for households of women (aged 15–49) and men (aged 15–54). The KDHS 2014 is the fifth demographic health survey in Kenya. It covered 36430 households, from which a total of 31079 women were interviewed, including 11555 women aged 15–24. Data were extracted for this group, and it emerged that 8560 of these women reported not using any method of contraception at the time of the survey; thus, they were excluded from the sample. From the remaining 2995, another 13 did not use a modern method of contraception and were also excluded. The selected sample of 2982 comprised women aged 15–24 who reported current use of any of the methods of modern contraception at the time of the survey, regardless of their marital status. The inclusion criterion was the use of modern contraception, while the non-use of contraception/modern methods resulted in exclusion. Specifically, data were obtained from the contraceptive calendar contained in the questionnaire for all interviewed women aged 15–24 within the individual women recode files.

### KDHS sampling

The KDHS 2014 used a stratified sample drawn from a national master sample, NASSEP V. This contained 5360 clusters split into 4 equal sub-samples that were broken down into 96,251 enumeration areas, and were then split further into households spread across the 47 counties in the country. The counties were stratified into urban and rural stratums. The KDHS 2014 sample targeted 40,300 households from 1612 clusters from around the country. More details on the KDHS’ 2014 process of sampling, data collection, and analysis, as well as the variables for which data were collected, are available online [6].

### Data variables

The dependent/outcome variable in the study was the current method of contraception (V312), which was coded as 1 if using LARC (IUD or implants) and 0 if using another modern method (contraceptive pill, male/female condom, injection, female sterilisation, periodic abstinence, or withdrawal method). It was obtained from two KDHS questions: ‘Are you currently doing something or using any method to delay or avoid getting pregnant?’ and ‘Which method are you using?’

Independent variables were selected from household- and female-level characteristics. The household-level variables were wealth status (V190), residence (V025), and region (V024), while the female-level variables were age (V013), education (V106), marital status (V501), religion (V130), number of living children (V218), and desire for children in the future (V605). Most of the variables were recoded to suit the focus of the study. Education was re-coded into none/primary and secondary/higher categories, while wealth status was classified into three tertiles (lower, middle, and higher) to represent the KDHS categories of poorest/poorer, middle, and richer/richest, respectively. Region was presented as eight individual regions coded 0–7, and was also reclassified into two groups labelled high contraceptive use (Central region, Nairobi, and Eastern region) and low contraceptive use (the other five regions) to take into account the targeted family planning interventions based on regional contraceptive prevalence. Marital status was recoded into married or not married from the various categories, desire for children in the future was coded as those who wanted or did not want children, and the number of living children was categorised as none, 1–2 children, and 3+ children. Religion was coded as none/other, Catholic, Protestant/other Christian, and Muslim. Age and residence were retained in their original categories. The demographic health survey contains much information on the demand side for family planning, but little information is available on the supply side. Therefore, no variables were available in the data set to assess supply.

### Data analysis

The first step was to extract a dataset of women aged 15–24 from the larger dataset of all interviewed women aged 15–49 and then to determine the frequencies of the current contraceptive methods used by the women at the time of the survey for inclusion or exclusion into the sample. Next, this study profiled young women based on the characteristics of the selected independent variables using cross-classification analysis. Bivariate analysis was conducted to establish the differentials in the use of LARC based on the different independent variables, and Pearson’s Chi-Squared test (χ^2^) was used to determine statistical significance in the selected variables against the use of LARC. The confidence level was set at 95% and significance at *p* < 0.05. All the data was weighted using the recommended DHS weighting system for individual women data obtained by weight = v005/1000000.

The dependent variable had two categories (LARC and other modern methods), and a regression model was selected to determine whether the independent variables had any effect on the current contraceptive method using binary logistic regression. The outcome of interest for this study was LARC usage, hence it was the reference category in the regression analysis. SPSS Version 22 was used to analyse the data.

## Results

### Characteristics of the study population

The first stage of the analysis profiled the study group against the selected variable characteristics, and the results are presented in Table 1.

**Table 1:** Distribution of women aged 15–24 using modern methods of contraception in Kenya, 2014 via background characteristics.

Variable	Number (weighted)	%
**Age**		
15–19	587	19.7
20–24	2395	80.3
**Education**		
None/primary	1488	49.9
Secondary +	1494	50.1
**Residence**		
Urban	1442	48.4
Rural	1540	51.6
**Wealth**		
Lower	890	29.9
Middle	625	21.0
Higher	1466	49.1
**Region**		
High contraception	1239	41.6
Low contraception	1743	58.4
**Region (Individual)**		
North-Eastern	2	0.1
Coast	246	8.3
Eastern	449	15.1
Central	342	11.5
Rift Valley	777	26.1
Western	289	9.7
Nyanza	428	14.4
Nairobi	448	15.0
**Religion**		
None/other	33	1.1
Catholic	659	22.1
Protestant/other Christian	2185	73.4
Muslim	100	3.4
**Marital Status**		
Married/living together	1945	65.2
Not married/not living together	1037	34.8
**No. of Living Children**		
None	594	19.9
1–2	2107	70.7
3+	281	9.4
**Desire for More Children**		
Want	1128	37.8
Do not want	1854	62.2
**Total *N***	**2982**	**100**

Source: KDHS 2014 [6]

Analysis of the background characteristics revealed that the majority (80.3%) of this study’s population were aged 20–24. For the education variable, the number of those who had either no education or primary education was almost equal to those who had secondary education. A similar picture emerged for residence, with 51.6% of the population living rurally. For wealth, the majority were from higher-wealth households (49.1%), while women from low contraception regions were a majority (58.4%). In terms of individual regions, Rift Valley, the Eastern region, and Nairobi had the most female users of modern contraception at 26.1%, 15.1%, and 15.0%, respectively.

The majority of women were married or were living with a partner (65.2%), and 70.7% had one or two children, while 9.4% had at least three children. Those with no children constituted 19.9%, while the majority (62.2%) did not want more children in the future. For religion, the Protestant/other Christian category were the majority at 73.4%.

### Differentials in LARC use

Cross-tabulations were performed to show any statistical associations between the variables under study against the two categories of LARC and other modern methods. The results are presented in Table 2.

**Table 2:** Differentials of LARC use for women aged 15–24 in Kenya, 2014 via background characteristics.

Variable	LARC	Other Modern Method	Total *N*	
	Number (%) (weighted)	Number (%) (weighted)	Number (%) (weighted)	χ^2^ *p*-value
**Age**				0.000
15–19	73 (12.5)	513 (87.5)	586 (19.7)	
20–24	463 (19.3)	1932 (80.7)	2395 (80.3)	
**Education**				0.022
None/primary	292 (19.6)	1196 (80.4)	1488 (49.9)	
Secondary +	247 (16.4)	1249 (83.6)	1494 (50.1)	
**Residence**				0.002
Urban	290 (20.1)	1152 (79.9)	1442 (48.4)	
Rural	247 (16.0)	1293 (84.0)	1540 (51.6)	
**Wealth**				0.624
Lower	151 (17.0)	739 (83.0)	890 (29.9)	
Middle	117 (18.7)	508 (81.3)	625 (21.0)	
Higher	268 (18.3)	1198 (81.7)	1466 (49.1)	
**Region**				0.020
High contraception	199 (16.1)	1040 (83.9)	1239 (41.5)	
Low contraception	338 (19.4)	1405 (80.6)	1743 (58.5)	
**Region (Individual)**				0.000
North-Eastern	1 (50.0)	1 (50.0)	2 (0.1)	
Coast	70 (28.5)	176 (71.5)	246 (8.3)	
Eastern	53 (11.8)	396 (88.2)	449 (15.1)	
Central	67 (19.6)	275 (80.4)	342 (11.5)	
Rift Valley	78 (10.0)	699 (90.0)	777 (26.1)	
Western	80 (27.7)	209 (72.3)	289 (9.7)	
Nyanza	108 (25.2)	320 (74.8)	428 (14.4)	
Nairobi	79 (17.6)	369 (82.4)	448 (15.0)	
**Religion**				0.000
None/other	11 (33.3)	22 (66.7)	33 (1.1)	
Catholic	121 (18.4)	538 (81.6)	659 (22.1)	
Protestant/other Christian	371 (17.0)	1814 (83.0)	2185 (73.4)	
Muslim	33 (33.0)	67 (67.0)	100 (3.4)	
**Marital Status**				0.001
Married/living together	383 (19.7)	1562 (80.3)	1945 (65.2)	
Not married /not living together	153 (14.8)	883 (85.2)	1036 (34.8)	
**No. of Living Children**				0.000
None	11 (1.9)	583 (98.1)	594 (19.9)	
1–2	446 (21.2)	1661 (78.8)	2107 (70.7)	
3+	79 (28.1)	202 (71.9)	281 (9.4)	
**Desire for More Children**				0.002
Want	171 (15.2)	956 (84.8)	1127 (37.8)	
Do not want	365 (19.7)	1489 (80.3)	1854 (62.2)	
**Total *N***	**537 (18.0)**	**2445 (82.0)**	**2982 (100)**	

Source: KDHS 2014 [6]

Chi-Squared test; *p* < 0.05

Age showed a strongly significant association with LARC use. LARC use among adolescents (aged 15–19) was low at 12.5%, as expected, while other modern methods accounted for 87.5%. Similarly, the majority (80.7%) of those aged 20–24 used other modern methods, while LARC took a 19.3% share.

Education exhibited a significant relationship with LARC use. LARC users with no education or primary education accounted for 19.6%, versus 80.4% for other modern method users, while LARC users with secondary education accounted for 16.4%, versus 83.6% for other modern method users.

Residence showed a statistically significant association with LARC use, at 20.1% among urban dwellers and 16.0% among rural dwellers. Urban modern method users accounted for 79.9%, while rural users accounted for 84.0%.

Wealth showed no significant association with LARC use. The distribution of LARC users against wealth status accounted for 17.0% for the lower, 18.7% for the middle, and 18.3% for the higher wealth categories. Thus, the proportions of LARC users was about the same across the different wealth tertiles. For the other modern method users, 83.0% were from the lower wealth category, while the middle and higher wealth users had almost equal shares of 81.3% and 81.7%, respectively.

Region had a significant influence on LARC use at the individual region level and the aggregated level. There was more LARC use in the low contraception regions (19.4%) than in the high contraceptive regions (16.1%). For LARC use at the individual level, the Coast, Western region, and Nyanza accounted for 28.5%, 27.7%, and 25.2%, respectively. The North-Eastern region had the lowest LARC users in terms of actual numbers.

A significant association was established with LARC use and religion. Those with no religion/other or who were Muslim constituted the largest proportion of LARC users at 33.3%, Protestants and other Christians the smallest at 17.0%. Protestants/other Christians and Catholics led the usage of other modern methods at over 80.0% each, while women with no religion and those who were Muslim accounted for 67.0% each.

Marital status was a significant factor in the use of LARC, with 19.7% of users being married and 80.3% using other modern methods. In the unmarried category, 14.8% used LARC, while 85.2% used other modern methods. There was slightly higher LARC use among married adolescent girls and young women.

The number of living children showed a significant association with LARC use. LARC use was negligible (1.9%) among those with no children (98.1%) who used other modern methods. LARC use for women who had 1–2 children was 21.2% against the use of other modern methods at 78.8%. For those with 3+ children, LARC use accounted for 28.1%, while other modern methods accounted for 71.9%.

Statistical significance was also seen in the desire for more children, as 15.2% of those who wanted more children were LARC users, while 84.8% used other modern methods.

### Determinants of LARC use

During this stage of the analysis, all the variables were fitted into the regression model with LARC as the reference category for contraceptive use, the dependent variable. Reference categories for the independent variables were indicated for each variable. In the first model, the region was fitted in individual categories together with the variable of religion. Table 3 presents the results of Model 1.

**Table 3:** Logistic regression estimates for use of LARC among women aged 15–24 in Kenya, 2014.

Variable	*p*-value	Odds Ratio (OR)	95% Confidence Interval (CI)
**Age**			
15–19	0.039	0.735*	[0.549–0.984]
20–24	[Ref]	[Ref]	[Ref]
**Education**			
None/primary	[Ref]	[Ref]	[Ref]
Secondary +	0.373	1.103	[0.889–1.367]
**Residence**			
Urban	[Ref]	[Ref]	[Ref]
Rural	0.002	0.674*	[0.525–0.865]
**Wealth**			
Lower	[Ref]	[Ref]	[Ref]
Middle	0.354	1.146	[0.859–1.528]
Higher	0.215	1.199	[0.900–1.598]
**Region (Individually Coded)**			
North-Eastern	[Ref]	[Ref]	[Ref]
Coast	0.766	0.606	[0.023–16.262]
Eastern	0.441	0.274	[0.010–7.363]
Central	0.670	0.490	[0.018–13.143]
Rift Valley	0.354	0.212	[0.008–5.662]
Western	0.880	0.775	[0.029–20.813]
Nyanza	0.831	0.700	[0.026–18.702]
Nairobi	0.530	0.348	[0.013–9.333]
**Religion**			
None/other religion	[Ref]	[Ref]	[Ref]
Catholic	0.066	0.460	[0.201–1.051]
Protestant/other Christian	0.017	0.377*	[0.168–0.842]
Muslim	0.527	0.748	[0.305–1.837]
**Marital Status**			
Married/living together	0.013	0.746*	[0.592–0.940]
Not married/ not living together	[Ref]	[Ref]	[Ref]
**No. of Living Children**			
None	[Ref]	[Ref]	[Ref]
1–2	0.000	17.624*	[9.482–32.756]
3+	0.000	23.531*	[11.751–47.119]
**Desire for More Children**			
Want	0.131	0.849	[0.686–1.050]
Do not want	[Ref]	[Ref]	[Ref]

Source: KDHS 2014 [6]

Ref = Reference Category; *p <* 0.05

* = *p <* 0.05

From the regression results in the first model, five variables showed significant associations with LARC use: age, residence, religion, marital status, and number of living children. For the age variable, adolescent girls aged 15–19 had reduced odds of using LARC and were 27% less likely to use it than women aged 20–24 [OR = 0.735, CI = 0.549–0.984]. Residence showed a very strongly negative, significant association, as rural young women were 33% less likely to use LARC against modern methods than their urban counterparts [OR = 0.674, CI = 0.525–0.865].

Religion was another significant predictor of LARC use. Protestant and other Christian women were about 63% less likely to use LARC than those with no religion/other religion [OR = 0.377, CI = 0.168–0.842].

Marital status had a moderate negative influence on LARC use. Married young women were 26% less likely to use LARC than their counterparts who were not married or living with a partner [OR = 0.746, CI = 0.592–0.940]. The number of living children showed the strongest positive relationship among all the independent variables, revealing that the more children a woman has, the more likely she is to choose LARC. Young women with up to 2 living children were about 18 times more likely to choose LARC than those who had no living children [OR = 17.624, CI = 9.482–32.756]. To affirm this relationship, those with at least 3 living children were shown to be 26 times more likely to use LARC than their counterparts with no living children [OR = 25.531, CI = 11.751–47.119].

A second regression model was fitted where religion was omitted and region was fitted as two aggregated categories (labelled high contraceptive and low contraceptive). The results of Model 2 are presented in Table 4.

**Table 4:** Logistic regression estimates for use of LARC among women aged 15–24 in Kenya, 2014.

Variable	*p*-value	Odds Ratio (OR)	95% Confidence Interval (CI)
**Age**			
15–19	0.161	0.817	[0.616–1.084]
20–24	[Ref]	[Ref]	[Ref]
**Education**			
None/primary	[Ref]	[Ref]	[Ref]
Secondary +	0.420	1.089	[0.885–1.340]
**Residence**			
Urban	[Ref]	[Ref]	[Ref]
Rural	0.000	0.625*	[0.496–0.789]
**Wealth**			
Lower	[Ref]	[Ref]	[Ref]
Middle	0.345	1.143	[0.866–1.510]
Higher	0.466	1.107	[0.842–1.455]
**Region (Contraception)**			
High contraception	0.017	0.773*	[0.626–0.955]
Low contraception	[Ref]	[Ref]	[Ref]
**Marital Status**			
Married/living together	0.008	0.738*	[0.589–0.923]
Not married/not living together	[Ref]	[Ref]	[Ref]
**No. of Living Children**			
None	[Ref]	[Ref]	[Ref]
1–2	0.000	17.197*	[9.274–31.887]
3+	0.000	25.767*	[12.967–51.201]
**Desire for More Children**			
Want	0.174	0.866	[0.703–1.066]
Do not want	[Ref]	[Ref]	[Ref]

Source: KDHS 2014 [6]

Ref = Reference Category; *p* < 0.05

* = *p <* 0.05

The results show that four variables (residence, region, marital status, and the number of living children) emerged as predictors of LARC use. Residence had a significant influence on LARC use. Rural women were 38% less likely to use it than their urban counterparts [OR = 0.625, CI = 0.496–0.789]. Women from high contraceptive regions were also found to be 23% less likely to do so than those from low contraceptive regions [OR = 0.773, CI = 0.626–0.955].

Marital status exhibited a predictive influence on LARC use, and married women were 27% less likely to use LARC than their unmarried counterparts [OR = 0.738, CI = 0.589–0.923]. The number of living children again emerged as a strong predictor of LARC use. Women with 1–2 children were 17 times more likely to use LARC than those with no children [OR = 17.197, CI = 9.274–31.887], while women with 3+ children were 26 times more likely to use it than nulliparous women [OR = 25.767, CI = 12.967–51.201].

## Discussion

This study focused on the use of LARC in comparison to other modern methods of contraception. The results showed that 18% of adolescents and young women used LARC. The results are in agreement with previous studies, as LARC use among the study group only began to increase in the last decade. It was not previously encouraged for this age group, as they had not attained their desired family size. However, LARC has recently been recommended and promoted for adolescents and women with no children, hence the increase in usage [29–31].

Factors that showed statistical significance at the cross-tabulation level were age, residence, region (individually and grouped), religion, marital status, the number of living children, and desire for children. Wealth, education, individual regions, and desire for children in the future did not exhibit any influence in the regression analysis, while predictors of LARC use emerged as age, residence, region (aggregated), religion, marital status, and number of living children.

For age, as expected, there was more LARC usage among those aged 20–24. This group is more mature and more exposed to sexual relationships, as they could be entering long-term relationships or marriage since the mean age of first marriage is 20 years old in Kenya [6]. Age had a significant and direct impact on LARC use because of the varied perceptions of its suitability for women aged 15–24, the indirect effects from exposure and access to contraception information, and LARC’s links with a woman’s parity and family planning intentions. It is an explanatory variable for contraceptive use in Kenya [9, 27].

By residence, more LARC users were found in urban areas, suggesting greater access and exposure to LARC methods. This variable emerged as a strong predictor of LARC use in rural areas, and points to the continued challenge of the availability of LARC services in rural areas, especially for the study group. LARC depends on provider skills as it involves insertions and removals, and these results may point to a shortage of skilled providers in rural areas. On the other hand, provider bias against young women could exist where LARC information and methods are not being provided to them because of their age. A previous national study in Kenya [32] similarly found the probability of using long-term methods to be higher in urban areas, and the results of this present study suggest that inequalities in service provisions remain. Recent studies have also found a higher prevalence of LARC use in urban areas in Eastern Africa [27].

Region also showed a significant influence on LARC use at the aggregated level, but not at the individual level. When individual regions were regressed together with religion, the effects of region were overshadowed, but when religion was controlled for, region was significant at the grouped level of high and low contraception. It was interesting to see that there was more LARC use in regions with low contraception, which might suggest the success of the LARC promotional campaigns in the last decade that have been spearheaded by the government, especially the family planning programme and the launch of FP2020. As a result, there has been increased usage of modern methods in Kenya, especially in areas where contraception is lower, thus increasing the use of LARC and more equity in terms of its access [32]. The use of long-term methods was more prevalent in regions of high contraception, while regional differentials were identified by a previous study [33].

Religion emerged as an explanatory variable for LARC use. Indeed, religion is deeply rooted in Kenya, and the different beliefs/practices influence the choice of contraception for some women. Other studies have documented the influence of Christianity and the specifically reduced odds of LARC use among Protestant Christians [34].

In the bivariate results on marital status, there was more LARC usage among married women, who were obviously more exposed to the risk of pregnancy and were faced with the choice of either spacing or limiting children. This was expected since married women may intend to start bearing children soon and thus might oppose LARC use, which is long-term and could delay fertility. Instead, they may choose short-acting methods that suit their pregnancy intentions.

However, there was greater use of other modern methods among unmarried women, suggesting an increase in sexual relationships and risk of unintended pregnancies among this group. This translates into a potential for greater LARC use among this group as they continue to move away from short-term methods. Marital status was shown to exert a negative influence on LARC in congruence with other studies of young women [35].

For the number of living children, there was, as expected, more LARC use among those with children, especially those with at least three children. This study found that there was more LARC use among those who did not want more children (limiters), suggesting that young women are using methods that meet their reproductive health goals. Women have around four children on average in Kenya, which this study’s findings corroborate [6]. The number of living children emerged as having a strong positive influence on LARC use. The fact that the odds of using LARC increased with the rise in the number of children may also suggest the successful integration of family planning/maternal child health programmes, for when women use these services, they are exposed to more LARC information and services and can consider meeting their demand for spacing or limiting children. Recent studies in Kenya have documented the success of such integration efforts [36, 37].

The fact that wealth showed no significant effect on LARC use at any level of the analysis might suggest success in the supply of contraception, as driven by the FP2020 campaign alongside the Ministry of Health, so that issues of cost have been eliminated and LARC is freely available to all potential users [38]. Previous studies in Kenya have found wealth to positively influence contraception use [33, 39].

Education showed no significance at any level of the analysis, which is in agreement with other recent studies in Kenya [40]. It appears that better information and exposure to contraceptive services associated with increased education has been overshadowed by improved information, access, and availability for all potential users in the promotion of LARC methods. These findings suggest the waning influence of socio-economic factors of education and wealth on LARC use for these young women.

The rise in the use of implants may be attributed to the success in the synergy of efforts by the government through the Ministry of Health and other partners to promote the use of LARC in Kenya from around 2010, and more so after the development of the CIP in 2012–2016 [41]. The Implant Access programme led by Marie Stopes [42], the Tupange programme funded by the Bill and Melinda Gates Foundation [43], the Tunza Clinic Programme by Population Services International [44], and FP2020 have each worked to generate demand for modern contraception in the areas that were lagging in prevalence. These efforts resulted in the supply of more than 1.8 million implants over about 5 years, and a huge increase in their uptake [42, 45]. The National Council for Population and Development also relaunched advocacy for family planning by emphasising the small family norm in 2011 and the Population Policy for National Development in 2012. To meet the demand, there is a need to improve the environment for commodity availability and supply. The supply chain shifted from the Ministry of Health to the Kenya Medical Supplies Agency, and private sector and non-governmental organisation commodity suppliers were used to cater to adolescents who receive supplies from pharmacies. Commodity budgets were also increased and were ring-fenced [41].

To address the reproductive health needs of youth, interventions targeting underserved populations such as adolescents and youth were designed, and youth-friendly services were established in health facilities. Community distribution was revitalised through community health workers to widen the programme’s reach. The National Adolescent Sexual and Reproductive Health policy was developed by the Ministry of Health in 2015 to give direction to adolescent-targeted programme efforts [41, 46].

### Implications for Kenya

The findings of this study hold some policy and programmatic implications. In general, it seems that adolescent girls and young women are realising the benefits of LARC, which spells some success for youth-targeted programmes. However, over 80% of those who did not want children in the future were not using LARC, and they should be targeted, as LARC provides highly effective protection against unintended pregnancies. More adolescent and youth-targeted programmes should be designed to encourage its uptake among non-users. Hopefully, the programmes can reduce the rates of unwanted pregnancy by reducing non-use, incorrect use, and inconsistent use of contraception, which is common in this group. The rising demand for LARC among adolescent girls and young women suggests the need for enhanced counselling to heighten their knowledge of the methods and to manage their expectations to reduce premature discontinuation. Counselling to reduce misconceptions and fears over the side effects of LARC should be a key part of targeted reproductive health programmes. Providers also need to emphasise to these young clients, especially to those without children, that the methods are reversible and that fertility returns soon after removal.

Regular and refresher training is critical for LARC service providers in terms of insertion and removal to improve services in rural areas and to ensure equitable access and availability within urban areas. Training should address possible provider bias by emphasising that LARC methods are not only suitable but recommended for young, unmarried, and nulliparous women.

The shift seen in increased LARC use in previous lower contraceptive regions calls for both sustained LARC promotion in those regions and also for reinstating campaigns in regions of higher contraceptive use to protect the earlier gains in LARC use.

## Conclusion

This study establishes that LARC use is rising among adolescent girls and young women. Therefore, there is potential to increase its uptake by addressing the predictors of its use, which were identified herein as age, residence, type of contraceptive region, religion, marital status, and the number of living children. Barriers based on these factors should be addressed, and investments in quality family planning services should be made so that the high rates of unintended pregnancies may be eliminated and adolescent girls and young women can have control over their reproductive and life goals. Therefore, more knowledge of LARC and its benefits is needed among this critical segment of the population.

Twenty-five years of the ICPD’s programme of action and eight years of the FP2020 have brought about many achievements in terms of increased modern contraceptive uptake in Kenya, but the positive results are only just emerging for adolescent girls and young women. Against the backdrop of the ICPD25’s commitments and the final years of the FP2020, accelerated efforts are needed in Kenya’s march towards the five zeros of unmet need for contraception, teenage pregnancies, unsafe abortions, preventable maternal deaths, and preventable neonatal/infant deaths. LARC can achieve these for adolescents and young women and thus reduce the adverse social effects of unintended pregnancies, such as lost schooling. LARC should be promoted as a pathway towards better reproductive health for adolescent girls and young women whose numbers and unmet need for contraception can push the momentum for LARC use and modern contraception in Kenya.

## Supporting information

S1 Material Results outputs file. (PDF)
