# Supplementary material for: Use of long-acting reversible contraception among adolescents and young women in Kenya
Source: PLoS One. 2020 Nov 10;15(11):e0241506. doi: 10.1371/journal.pone.0241506 (PMC7654813; doi:10.1371/journal.pone.0241506)
Supplement: S1 Material — (PDF) [file pone.0241506.s001.pdf]

```
GET
  FILE='C:\Users\Wambui Kungu\Desktop\2014 KDHS Data Set.sav' /keep caseid v000
v001 v002 v003 v004 v005 v007 v008 v011 v012 v013 v021 v023 V025 V024 V106 V190
v201 v218 v501 v605 v106 v130 v190 v312 v313  V3A02 V3A03 V3A04 V3A05.

SELECT IF (V013 le 2).
FREQUENCIES VARIABLE = v312.
```

Statistics

Current contraceptive method

N	Valid	11555
	Missing	0

Current contraceptive method

		Frequency	Percent	Valid Percent	Cumulative Percent
Valid	Not using	8560	74.1	74.1	74.1
	Pill	298	2.6	2.6	76.7
	IUD	54	.5	.5	77.1
	Injections	1467	12.7	12.7	89.8
	Condom	429	3.7	3.7	93.5
	Female sterilization	3	.0	.0	93.5
	Periodic abstinence	205	1.8	1.8	95.3
	Withdrawal	35	.3	.3	95.6
	Other	9	.1	.1	95.7
	Implants/Norplant	483	4.2	4.2	99.9
	Lactational amenorrhea (LAM)	0	.0	.0	99.9
	Female condom	10	.1	.1	100.0
	Oher modern method	3	.0	.0	100.0
	Total	11555	100.0	100.0	

```
/ORDER=ANALYSIS

Error # 1.  Command name: /ORDER
The first word in the line is not recognized as an SPSS Statistics command.
Execution of this command stops.

SELECT IF
(v312=1|v312=2|v312=3|v312=4|v312=5|v312=6|v312=7|v312=8|v312=9|v312=11|v312=1
4|v312=15).
```

```
RECODE v312 (2,11=1) (1,3,4,5,6,7,8,9,14,15=0) into v312r.
VARIABLE LABELS v312r "Use LARC".
VALUE LABELS v312r 1 "Use LARC" 0 "Other Modern".

RECODE v106 (0,1=1) (2,3=2) into v106r.
VARIABLE LABELS v106r "Education recoded".
VALUE LABELS v106r 1 "None/Pri" 2"Sec/High".

RECODE V190 (1,2=1) (3=2) (4,5=3) into v190r.
VARIABLE LABELS v190r "Wealth status".
Value labels v190r 1 "Lower" 2 "Middle" 3 "Higher".

RECODE v218 (0=0) (1,2=1) (3 thru hi=2) into v218r.
VARIABLE LABELS V218r "Living children".
VALUE LABELS v218r 0 "No children" 1 "1-2 children" 2 "3+children".

RECODE v024 (2=0) (1=1) (3=2) (4=3) (5=4) (7=5) (8=6) ( 9=7) into v024r.
VARIABLE LABELS v024r "Region recoded".
VALUE LABELS v024r 0 "N Eastern" 1 "Coast" 2 "Eastern" 3 "Central" 4 "Rift Valley"
5 "Western" 6 "Nyanza" 7 "Nairobi".

FREQUENCIES VARIABLE = v024r.
```

Statistics

Region recoded

N	Valid	2982
	Missing	0

Region recoded

		Frequency	Percent	Valid Percent	Cumulative Percent
Valid	N Eastern	2	.1	.1	.1
	Coast	246	8.3	8.3	8.3
	Eastern	449	15.1	15.1	23.4
	Central	342	11.5	11.5	34.8
	Rift Valley	777	26.1	26.1	60.9
	Western	289	9.7	9.7	70.6
	Nyanza	428	14.4	14.4	85.0
	Nairobi	448	15.0	15.0	100.0
	Total	2982	100.0	100.0	

```
/ORDER=ANALYSIS

Error # 1.  Command name: /ORDER
```

The first word in the line is not recognized as an SPSS Statistics command.
Execution of this command stops.

```
RECODE v024 (3=1) (4=1) ( 9=1) (1=2) (2=2) (5=2) (7=2) (8=2) into v024g.
VARIABLE LABELS v024g "Regiong grouped recoded".
VALUE LABELS v024g 1 "High Contraception" 2 "Low Contraception" .

RECODE  v130 (4=0) (96=0) (1=1) (2=2) ( 3=3)  into v130r.
VARIABLE LABELS  v130r 'Religion'.
VALUE LABELS v130r 0 "None/other" 1 "Catholic" 2 "Protestant/other christian" 3
"Muslim".

RECODE v501 (1,2=1) (else=2) into v501r.
VARIABLE LABELS v501r "Marital status".
VALUE LABELS v501r 1 "Mar/ living together" 2 "Not married/Not Living Together".

RECODE v605 (1,2,3=1) (else=2) into v605r.
VARIABLE LABELS v605r "Desire for children".
VALUE LABELS v605r 1 "Wants" 2 "Do not want".

FREQUENCIES  V013 V024r V024g V025 v106r v130r v190r v218r v501r v605r v312r.
```

Statistics

		Age in 5-year groups	Region recoded	Regiong grouped recoded	Type of place of residence	Education recoded
N	Valid	2982	2982	2982	2982	2982
	Missing	0	0	0	0	0

Statistics

		Religion	Wealth status	Living children	Marital status	Desire for children
N	Valid	2977	2982	2982	2982	2982
	Missing	5	0	0	0	0

Statistics

		Use LARC
N	Valid	2982
	Missing	0

Frequency Table

Age in 5-year groups

		Frequency	Percent	Valid Percent	Cumulative Percent
Valid	15-19	587	19.7	19.7	19.7
	20-24	2395	80.3	80.3	100.0
	Total	2982	100.0	100.0	

Region recoded

		Frequency	Percent	Valid Percent	Cumulative Percent
Valid	N Eastern	2	.1	.1	.1
	Coast	246	8.3	8.3	8.3
	Eastern	449	15.1	15.1	23.4
	Central	342	11.5	11.5	34.8
	Rift Valley	777	26.1	26.1	60.9
	Western	289	9.7	9.7	70.6
	Nyanza	428	14.4	14.4	85.0
	Nairobi	448	15.0	15.0	100.0
	Total	2982	100.0	100.0	

Regiong grouped recoded

		Frequency	Percent	Valid Percent	Cumulative Percent
Valid	High Contraception	1239	41.6	41.6	41.6
	Low Contraception	1743	58.4	58.4	100.0
	Total	2982	100.0	100.0	

Type of place of residence

	Frequency	Percent	Valid Percent	Cumulative Percent

Valid	Urban	1442	48.4	48.4	48.4
	Rural	1540	51.6	51.6	100.0
	Total	2982	100.0	100.0	

Education recoded

		Frequency	Percent	Valid Percent	Cumulative Percent
Valid	None/Pri	1488	49.9	49.9	49.9
	Sec/High	1494	50.1	50.1	100.0
	Total	2982	100.0	100.0	

Religion

		Frequency	Percent	Valid Percent	Cumulative Percent
Valid	None/other	33	1.1	1.1	1.1
	Catholic	659	22.1	22.1	23.2
	Protestant/other christian	2185	73.3	73.4	96.6
	Muslim	100	3.4	3.4	100.0
	Total	2977	99.8	100.0	
Missing	System	5	.2		
Total		2982	100.0		

Wealth status

		Frequency	Percent	Valid Percent	Cumulative Percent
Valid	Lower	890	29.9	29.9	29.9
	Middle	625	21.0	21.0	50.8
	Higher	1466	49.2	49.2	100.0
	Total	2982	100.0	100.0	

Living children

		Frequency	Percent	Valid Percent	Cumulative Percent
Valid	No children	594	19.9	19.9	19.9
	1-2 children	2107	70.7	70.7	90.6
	3+children	281	9.4	9.4	100.0
	Total	2982	100.0	100.0	

Marital status

		Frequency	Percent	Valid Percent	Cumulative Percent
Valid	Mar/ living together	1945	65.2	65.2	65.2
	Not married/Not Living Together	1037	34.8	34.8	100.0
	Total	2982	100.0	100.0	

Desire for children

		Frequency	Percent	Valid Percent	Cumulative Percent
Valid	Wants	1128	37.8	37.8	37.8
	Do not want	1854	62.2	62.2	100.0
	Total	2982	100.0	100.0	

Use LARC

		Frequency	Percent	Valid Percent	Cumulative Percent
Valid	Other Modern	2445	82.0	82.0	82.0
	Use LARC	537	18.0	18.0	100.0
	Total	2982	100.0	100.0	

```
GET
  FILE='C:\Users\Wambui Kungu\Desktop\2014 KDHS Data Set.sav' /keep caseid v000 v001 v002 v003
DATASET NAME DataSet1 WINDOW=FRONT.
```

Dataset Name

Warnings

The active dataset will replace the existing dataset named DataSet1.

```
COMPUTE weight=v005/1000000.
EXECUTE.
WEIGHT BY weight.
```

```
SELECT IF (V013 le 2).
FREQUENCIES VARIABLE = v312.
```

Frequencies

Statistics

Current contraceptive method

N	Valid	11555
	Missing	0

Current contraceptive method

		Frequency	Percent	Valid Percent	Cumulative Percent
Valid	Not using	8560	74.1	74.1	74.1
	Pill	298	2.6	2.6	76.7
	IUD	54	.5	.5	77.1
	Injections	1467	12.7	12.7	89.8
	Condom	429	3.7	3.7	93.5
	Female sterilization	3	.0	.0	93.5
	Periodic abstinence	205	1.8	1.8	95.3
	Withdrawal	35	.3	.3	95.6
	Other	9	.1	.1	95.7
	Implants/Norplant	483	4.2	4.2	99.9
	Lactational amenorrhea (LAM)	0	.0	.0	99.9
	Female condom	10	.1	.1	100.0
	Oher modern method	3	.0	.0	100.0
	Total	11555	100.0	100.0	

```
 /ORDER=ANALYSIS.

>Error # 1.  Command name: /ORDER
>The first word in the line is not recognized as an SPSS Statistics command.
>Execution of this command stops.
SELECT IF (v312=1|v312=2|v312=3|v312=4|v312=5|v312=6|v312=7|v312=8|v312=9|v312=11|v312=14|v312=

RECODE v312 (2,11=1) (1,3,4,5,6,7,8,9,14,15=0) into v312r.
VARIABLE LABELS v312r "Use LARC".
VALUE LABELS v312r 1 "Use LARC" 0 "Other Modern".
FREQUENCIES VARIABLE = v312r.
```

Frequencies

Statistics

Use LARC

N	Valid	2982
	Missing	0

Use LARC

		Frequency	Percent	Valid Percent	Cumulative Percent
Valid	Other Modern	2445	82.0	82.0	82.0
	Use LARC	537	18.0	18.0	100.0
	Total	2982	100.0	100.0	

```
 /ORDER=ANALYSIS.

>Error # 1.  Command name: /ORDER
>The first word in the line is not recognized as an SPSS Statistics command.
>Execution of this command stops.
RECODE v106 (0,1=1) (2,3=2) into v106r.
VARIABLE LABELS v106r "Education recoded".
VALUE LABELS v106r 1 "None/Pri" 2"Sec/High".

RECODE V190 (1,2=1) (3=2) (4,5=3) into v190r.
VARIABLE LABELS v190r "Wealth status".
Value labels v190r 1 "Lower" 2 "Middle" 3 "Higher".

RECODE v218 (0=0) (1,2=1) (3 thru hi=2) into v218r.
VARIABLE LABELS V218r "Living children".
VALUE LABELS v218r 0 "No children" 1 "1-2 children" 2 "3+children".

RECODE v024 (3=1) (4=1) ( 9=1) (1=2) (2=2) (5=2) (7=2) (8=2) into v024r.
VARIABLE LABELS v024r "Region recoded".
VALUE LABELS v024r 1 "High Contraception" 2 "Low Contraception" .

FREQUENCIES VARIABLE = v024r.
```

Frequencies

Statistics

Region recoded

N	Valid	2982
	Missing	0

Region recoded

		Frequency	Percent	Valid Percent	Cumulative Percent
Valid	High Contraception	1239	41.6	41.6	41.6
	Low Contraception	1743	58.4	58.4	100.0
	Total	2982	100.0	100.0	

```
 /ORDER=ANALYSIS

>Error # 1.  Command name: /ORDER
>The first word in the line is not recognized as an SPSS Statistics command.
>Execution of this command stops.

RECODE  v130 (4=0) (96=0) (1=1) (2=2) ( 3=3)  into v130r.
VARIABLE LABELS  v130r 'Religion'.
VALUE LABELS v130r 0 "None/other" 1 "Catholic" 2 "Protestant/other christian" 3  "Muslim".

RECODE v501 (1,2=1) (else=2) into v501r.
VARIABLE LABELS v501r "Marital status".
VALUE LABELS v501r 1 "Mar/ living together" 2 "Not married/Not Living Together".

RECODE v605 (1,2,3=1) (else=2) into v605r.
VARIABLE LABELS v605r "Desire for children".
VALUE LABELS v605r 1 "Wants" 2 "Do not want".

FREQUENCIES  V013 V024r V025 v106r v130r v190r v218r v501r v605r V3A02 V3A03 v312 v312r.
```

Frequencies

Statistics

		Age in 5-year groups	Region recoded	Type of place of residence	Education recoded	Religion
N	Valid	2982	2982	2982	2982	2977
	Missing	0	0	0	0	5

Statistics

		Wealth status	Living children	Marital status	Desire for children	Told about side effects
N	Valid	2982	2982	2982	2982	1025
	Missing	0	0	0	0	1957

Statistics

		Told about side effects by health or family planning worker	Current contraceptive method	Use LARC
N	Valid	501	2982	2982
	Missing	2481	0	0

Frequency Table

Age in 5-year groups

		Frequency	Percent	Valid Percent	Cumulative Percent
Valid	15-19	587	19.7	19.7	19.7
	20-24	2395	80.3	80.3	100.0
	Total	2982	100.0	100.0	

Region recoded

		Frequency	Percent	Valid Percent	Cumulative Percent
Valid	High Contraception	1239	41.6	41.6	41.6
	Low Contraception	1743	58.4	58.4	100.0
	Total	2982	100.0	100.0	

Type of place of residence

		Frequency	Percent	Valid Percent	Cumulative Percent
Valid	Urban	1442	48.4	48.4	48.4
	Rural	1540	51.6	51.6	100.0
	Total	2982	100.0	100.0	

Education recoded

		Frequency	Percent	Valid Percent	Cumulative Percent
Valid	None/Pri	1488	49.9	49.9	49.9
	Sec/High	1494	50.1	50.1	100.0
	Total	2982	100.0	100.0	

Religion

		Frequency	Percent	Valid Percent	Cumulative Percent
Valid	None/other	33	1.1	1.1	1.1
	Catholic	659	22.1	22.1	23.2
	Protestant/other christian	2185	73.3	73.4	96.6
	Muslim	100	3.4	3.4	100.0
	Total	2977	99.8	100.0	
Missing	System	5	.2		
Total		2982	100.0		

Wealth status

		Frequency	Percent	Valid Percent	Cumulative Percent
Valid	Lower	890	29.9	29.9	29.9
	Middle	625	21.0	21.0	50.8
	Higher	1466	49.2	49.2	100.0
	Total	2982	100.0	100.0	

Living children

		Frequency	Percent	Valid Percent	Cumulative Percent
Valid	No children	594	19.9	19.9	19.9
	1-2 children	2107	70.7	70.7	90.6
	3+children	281	9.4	9.4	100.0
	Total	2982	100.0	100.0	

Marital status

		Frequency	Percent	Valid Percent	Cumulative Percent
Valid	Mar/ living together	1945	65.2	65.2	65.2
	Not married/Not Living Together	1037	34.8	34.8	100.0
	Total	2982	100.0	100.0	

Desire for children

		Frequency	Percent	Valid Percent	Cumulative Percent
Valid	Wants	1128	37.8	37.8	37.8
	Do not want	1854	62.2	62.2	100.0
	Total	2982	100.0	100.0	

Told about side effects

		Frequency	Percent	Valid Percent	Cumulative Percent
Valid	No	500	16.8	48.8	48.8
	Yes	525	17.6	51.2	100.0
	Total	1025	34.4	100.0	
Missing	9	6	.2		
	System	1951	65.4		
	Total	1957	65.6		
Total		2982	100.0		

Told about side effects by health or family planning worker

		Frequency	Percent	Valid Percent	Cumulative Percent
Valid	No	464	15.6	92.7	92.7
	Yes	37	1.2	7.3	100.0
	Total	501	16.8	100.0	
Missing	9	5	.2		
	System	2476	83.0		
	Total	2481	83.2		
Total		2982	100.0		

Current contraceptive method

		Frequency	Percent	Valid Percent	Cumulative Percent
Valid	Pill	298	10.0	10.0	10.0
	IUD	54	1.8	1.8	11.8
	Injections	1467	49.2	49.2	61.0
	Condom	429	14.4	14.4	75.3
	Female sterilization	3	.1	.1	75.4
	Periodic abstinence	205	6.9	6.9	82.3
	Withdrawal	35	1.2	1.2	83.5
	Implants/Norplant	483	16.2	16.2	99.7
	Female condom	10	.3	.3	100.0
	Total	2982	100.0	100.0	

Use LARC

		Frequency	Percent	Valid Percent	Cumulative Percent
Valid	Other Modern	2445	82.0	82.0	82.0
	Use LARC	537	18.0	18.0	100.0
	Total	2982	100.0	100.0	

```
 /ORDER=ANALYSIS.

>Error # 1.  Command name: /ORDER
>The first word in the line is not recognized as an SPSS Statistics command.
>Execution of this command stops.
CROSSTABS
  /TABLES=v312r BY V013 V025 v106r v190r v218r v024r v501r v130r v605r
  /FORMAT=AVALUE TABLES
  /STATISTICS=CHISQ
  /CELLS=COUNT ROW
  /COUNT ROUND CELL.
```

Crosstabs

Case Processing Summary

	Cases					
	Valid		Missing		Total	
	N	Percent	N	Percent	N	Percent
Use LARC * Age in 5-year groups	2981	100.0%	.873	0.0%	2981.873	100.0%
Use LARC * Type of place of residence	2982[a]	100.0%	0	0.0%	2981.873	100.0%
Use LARC * Education recoded	2982[a]	100.0%	0	0.0%	2981.873	100.0%
Use LARC * Wealth status	2981[a]	100.0%	.873	0.0%	2981.873	100.0%
Use LARC * Living children	2982[a]	100.0%	0	0.0%	2981.873	100.0%
Use LARC * Region recoded	2982[a]	100.0%	0	0.0%	2981.873	100.0%
Use LARC * Marital status	2981[a]	100.0%	.873	0.0%	2981.873	100.0%
Use LARC * Religion	2977[a]	99.8%	4.873	0.2%	2981.873	100.0%
Use LARC * Desire for children	2981[a]	100.0%	.873	0.0%	2981.873	100.0%

a. Number of valid cases is different from the total count in the crosstabulation table because the cell counts have been rounded.

```
 LOGISTIC REGRESSION VARIABLES v312r
  /METHOD=ENTER V013 V025 v106r v190r v218r v024r v130r v501r v605r
  /CONTRAST (V013)=Indicator
  /CONTRAST (V025)=Indicator(1)
  /CONTRAST (v106r)=Indicator(1)
  /CONTRAST (v190r)=Indicator(1)
  /CONTRAST (v218r)=Indicator(1)
```

```
/CONTRAST (v024r)=Indicator
/CONTRAST (v130r)=Indicator(1)
/CONTRAST (v501r)=Indicator
/CONTRAST (v605r)=Indicator
/PRINT=GOODFIT SUMMARY CI(95)
/CRITERIA=PIN(0.05) POUT(0.10) ITERATE(20) CUT(0.5).
```

Logistic Regression

Case Processing Summary

Unweighted Cases[a]		N	Percent
Selected Cases	Included in Analysis	2554	99.8
	Missing Cases	5	.2
	Total	2559	100.0
Unselected Cases		0	.0
Total		2559	100.0

a. If weight is in effect, see classification table for the total number of cases.

Dependent Variable Encoding

Original Value	Internal Value
Other Modern	0
Use LARC	1

Categorical Variables Codings

		Frequency	Parameter coding (1)	(2)	(3)
Religion	None/other	33	.000	.000	.000
	Catholic	539	1.000	.000	.000
	Protestant/other christian	1845	.000	1.000	.000
	Muslim	137	.000	.000	1.000
Living children	No children	442	.000	.000	
	1-2 children	1815	1.000	.000	
	3+children	297	.000	1.000	
Wealth status	Lower	952	.000	.000	
	Middle	566	1.000	.000	
	Higher	1036	.000	1.000	
Type of place of residence	Urban	1128	.000		
	Rural	1426	1.000		
Education recoded	None/Pri	1398	.000		
	Sec/High	1156	1.000		
Desire for children	Wants	972	1.000		
	Do not want	1582	.000		
Marital status	Mar/ living together	1706	1.000		
	Not married/Not Living Together	848	.000		
Region recoded	High Contraception	791	1.000		
	Low Contraception	1763	.000		
Age in 5-year groups	15-19	517	1.000		
	20-24	2037	.000		

Block 0: Beginning Block

Classification Table[a,b]

Observed			Predicted: Use LARC: Other Modern	Use LARC	Percentage Correct
Step 0	Use LARC	Other Modern	2441	0	100.0
		Use LARC	536	0	.0
	Overall Percentage				82.0

a. Constant is included in the model.

b. The cut value is .500

Variables in the Equation

		B	S.E.	Wald	df	Sig.	Exp(B)
Step 0	Constant	-1.517	.048	1010.594	1	.000	.219

Variables not in the Equation

			Score	df	Sig.
Step 0	Variables	V013(1)	14.769	1	.000
		V025(1)	8.633	1	.003
		v106r(1)	5.036	1	.025
		v190r	.961	2	.618
		v190r(1)	.320	1	.572
		v190r(2)	.163	1	.687
		v218r	138.209	2	.000
		v218r(1)	48.474	1	.000
		v218r(2)	22.184	1	.000
		v024r(1)	5.230	1	.022
		v130r	21.441	3	.000
		v130r(1)	.073	1	.787
		v130r(2)	5.685	1	.017
		v130r(3)	15.705	1	.000
		v501r(1)	10.855	1	.001
		v605r(1)	9.619	1	.002
	Overall Statistics		195.267	13	.000

Block 1: Method = Enter

Omnibus Tests of Model Coefficients

		Chi-square	df	Sig.
Step 1	Step	245.473	13	.000
	Block	245.473	13	.000
	Model	245.473	13	.000

Model Summary

Step	-2 Log likelihood	Cox & Snell R Square	Nagelkerke R Square
1	2560.481[a]	.079	.130

a. Estimation terminated at iteration number 7 because parameter estimates changed by less than .001.

Hosmer and Lemeshow Test

Step	Chi-square	df	Sig.
1	19.960	8	.010

Contingency Table for Hosmer and Lemeshow Test

		Use LARC = Other Modern		Use LARC = Use LARC		Total
		Observed	Expected	Observed	Expected	
Step 1	1	291	293.389	7	4.183	298
	2	293	291.212	6	7.470	299
	3	247	257.365	49	39.171	297
	4	240	249.469	57	47.086	297
	5	261	244.141	37	54.090	298
	6	248	237.901	51	60.759	299
	7	249	245.476	67	70.563	316
	8	223	227.441	78	73.018	300
	9	225	217.187	74	81.570	299
	10	166	177.651	110	97.730	275

Classification Table[a]

Observed			Predicted		
			Use LARC		Percentage Correct
			Other Modern	Use LARC	
Step 1	Use LARC	Other Modern	2439	3	99.9
		Use LARC	528	8	1.4
	Overall Percentage				82.2

a. The cut value is .500

Variables in the Equation

		B	S.E.	Wald	df	Sig.	Exp(B)	95% C.I....
								Lower
Step 1[a]	V013(1)	-.253	.146	3.007	1	.083	.776	.583
	V025(1)	-.429	.119	12.900	1	.000	.651	.515
	v106r(1)	.113	.107	1.124	1	.289	1.120	.908
	v190r			1.362	2	.506		
	v190r(1)	.160	.143	1.254	1	.263	1.174	.887
	v190r(2)	.121	.141	.741	1	.389	1.129	.856
	v218r			89.140	2	.000		
	v218r(1)	2.849	.315	81.643	1	.000	17.276	9.312
	v218r(2)	3.274	.351	86.853	1	.000	26.415	13.269
	v024r(1)	-.211	.109	3.740	1	.053	.810	.654
	v130r			17.395	3	.001		
	v130r(1)	-.588	.407	2.093	1	.148	.555	.250
	v130r(2)	-.791	.396	3.996	1	.046	.454	.209
	v130r(3)	.048	.447	.011	1	.915	1.049	.436
	v501r(1)	-.311	.115	7.274	1	.007	.733	.585
	v605r(1)	-.147	.107	1.899	1	.168	.863	.700
	Constant	-2.988	.514	33.813	1	.000	.050	

Variables in the Equation

		95% C.I....
		Upper
Step 1[a]	V013(1)	1.034
	V025(1)	.823
	v106r(1)	1.381
	v190r	
	v190r(1)	1.554
	v190r(2)	1.488
	v218r	
	v218r(1)	32.052
	v218r(2)	52.587
	v024r(1)	1.003
	v130r	
	v130r(1)	1.232
	v130r(2)	.985
	v130r(3)	2.520
	v501r(1)	.919
	v605r(1)	1.064
	Constant	

a. Variable(s) entered on step 1: V013, V025, v106r, v190r, v218r, v024r, v130r, v501r, v605r.

```
LOGISTIC REGRESSION VARIABLES v312r
 /METHOD=ENTER V013 V025 v106r v190r v218r v024r v501r v605r
 /CONTRAST (V013)=Indicator
 /CONTRAST (V025)=Indicator(1)
 /CONTRAST (v106r)=Indicator(1)
 /CONTRAST (v190r)=Indicator(1)
 /CONTRAST (v218r)=Indicator(1)
 /CONTRAST (v024r)=Indicator
 /CONTRAST (v501r)=Indicator
 /CONTRAST (v605r)=Indicator
 /PRINT=GOODFIT SUMMARY CI(95)
 /CRITERIA=PIN(0.05) POUT(0.10) ITERATE(20) CUT(0.5).
```

Logistic Regression

Case Processing Summary

Unweighted Cases[a]		N	Percent
Selected Cases	Included in Analysis	2559	100.0
	Missing Cases	0	.0
	Total	2559	100.0
Unselected Cases		0	.0
Total		2559	100.0

a. If weight is in effect, see classification table for the total number of cases.

Dependent Variable Encoding

Original Value	Internal Value
Other Modern	0
Use LARC	1

Categorical Variables Codings

		Frequency	Parameter coding (1)	Parameter coding (2)
Wealth status	Lower	955	.000	.000
	Middle	566	1.000	.000
	Higher	1038	.000	1.000
Living children	No children	443	.000	.000
	1-2 children	1819	1.000	.000
	3+children	297	.000	1.000
Type of place of residence	Urban	1128	.000	
	Rural	1431	1.000	
Education recoded	None/Pri	1401	.000	
	Sec/High	1158	1.000	
Desire for children	Wants	972	1.000	
	Do not want	1587	.000	
Region recoded	High Contraception	792	1.000	
	Low Contraception	1767	.000	
Marital status	Mar/ living together	1710	1.000	
	Not married/Not Living Together	849	.000	
Age in 5-year groups	15-19	518	1.000	
	20-24	2041	.000	

Block 0: Beginning Block

Classification Table[a,b]

Observed			Predicted: Use LARC: Other Modern	Predicted: Use LARC: Use LARC	Predicted: Percentage Correct
Step 0	Use LARC	Other Modern	2445	0	100.0
		Use LARC	537	0	.0
	Overall Percentage				82.0

a. Constant is included in the model.

b. The cut value is .500

Variables in the Equation

		B	S.E.	Wald	df	Sig.	Exp(B)
Step 0	Constant	-1.516	.048	1011.767	1	.000	.220

Variables not in the Equation

			Score	df	Sig.
Step 0	Variables	V013(1)	14.977	1	.000
		V025(1)	8.525	1	.004
		v106r(1)	5.067	1	.024
		v190r	.925	2	.630
		v190r(1)	.310	1	.578
		v190r(2)	.155	1	.694
		v218r	138.653	2	.000
		v218r(1)	48.868	1	.000
		v218r(2)	22.121	1	.000
		v024r(1)	5.369	1	.020
		v501r(1)	11.070	1	.001
		v605r(1)	9.682	1	.002
	Overall Statistics		177.569	10	.000

Block 1: Method = Enter

Omnibus Tests of Model Coefficients

		Chi-square	df	Sig.
Step 1	Step	229.682	10	.000
	Block	229.682	10	.000
	Model	229.682	10	.000

Model Summary

Step	-2 Log likelihood	Cox & Snell R Square	Nagelkerke R Square
1	2582.008[a]	.074	.121

a. Estimation terminated at iteration number 7 because parameter estimates changed by less than .001.

Hosmer and Lemeshow Test

Step	Chi-square	df	Sig.
1	13.318	8	.101

Contingency Table for Hosmer and Lemeshow Test

		Use LARC = Other Modern		Use LARC = Use LARC		Total
		Observed	Expected	Observed	Expected	
Step 1	1	291	293.798	7	4.359	298
	2	294	292.097	6	7.334	299
	3	234	241.508	46	38.762	280
	4	206	211.933	47	41.340	253
	5	258	252.336	50	55.613	308
	6	278	265.814	56	68.380	334
	7	222	212.172	51	61.185	273
	8	215	222.796	78	70.824	294
	9	224	219.298	74	79.107	298
	10	221	233.245	122	109.971	343

Classification Table[a]

Observed			Predicted		
			Use LARC		Percentage Correct
			Other Modern	Use LARC	
Step 1	Use LARC	Other Modern	2445	0	100.0
		Use LARC	537	0	.0
	Overall Percentage				82.0

a. The cut value is .500

Variables in the Equation

		B	S.E.	Wald	df	Sig.	Exp(B)	95% C.I...
								Lower
Step 1[a]	V013(1)	-.202	.144	1.968	1	.161	.817	.616
	V025(1)	-.469	.119	15.669	1	.000	.625	.496
	v106r(1)	.085	.106	.650	1	.420	1.089	.885
	v190r			.972	2	.615		
	v190r(1)	.134	.142	.890	1	.345	1.143	.866
	v190r(2)	.102	.140	.530	1	.466	1.107	.842
	v218r			88.492	2	.000		
	v218r(1)	2.845	.315	81.534	1	.000	17.197	9.274
	v218r(2)	3.249	.350	86.006	1	.000	25.767	12.967
	v024r(1)	-.258	.108	5.708	1	.017	.773	.626
	v501r(1)	-.304	.115	7.057	1	.008	.738	.589
	v605r(1)	-.144	.106	1.847	1	.174	.866	.703
	Constant	-3.626	.346	109.682	1	.000	.027	

Variables in the Equation

		95% C.I....
		Upper
Step 1[a]	V013(1)	1.084
	V025(1)	.789
	v106r(1)	1.340
	v190r	
	v190r(1)	1.510
	v190r(2)	1.455
	v218r	
	v218r(1)	31.887
	v218r(2)	51.201
	v024r(1)	.955
	v501r(1)	.923
	v605r(1)	1.066
	Constant	

a. Variable(s) entered on step 1: V013, V025, v106r, v190r, v218r, v024r, v501r, v605r.

```
 RECODE v024 (2=0) (1=1) (3=2) (4=3) (5=4) (7=5) (8=6) ( 9=7) into v024r.
VARIABLE LABELS v024r "Region recoded".
VALUE LABELS v024r 0 "N Eastern" 1 "Coast" 2 "Eastern" 3 "Central" 4 "Rift Valley" 5 "Western"

FREQUENCIES VARIABLE = v024r.
```

Frequencies

Statistics

Region recoded

N	Valid	2982
	Missing	0

Region recoded

		Frequency	Percent	Valid Percent	Cumulative Percent
Valid	N Eastern	2	.1	.1	.1
	Coast	246	8.3	8.3	8.3
	Eastern	449	15.1	15.1	23.4
	Central	342	11.5	11.5	34.8
	Rift Valley	777	26.1	26.1	60.9
	Western	289	9.7	9.7	70.6
	Nyanza	428	14.4	14.4	85.0
	Nairobi	448	15.0	15.0	100.0
	Total	2982	100.0	100.0	

```
 /ORDER=ANALYSIS.

>Error # 1.  Command name: /ORDER
>The first word in the line is not recognized as an SPSS Statistics command.
>Execution of this command stops.
LOGISTIC REGRESSION VARIABLES v312r
  /METHOD=ENTER V013 V025 v106r v190r v218r v501r v605r v024r v130r
  /CONTRAST (V013)=Indicator
  /CONTRAST (V025)=Indicator(1)
  /CONTRAST (v106r)=Indicator(1)
  /CONTRAST (v190r)=Indicator(1)
  /CONTRAST (v218r)=Indicator(1)
  /CONTRAST (v501r)=Indicator
  /CONTRAST (v605r)=Indicator
  /CONTRAST (v024r)=Indicator(1)
  /CONTRAST (v130r)=Indicator(1)
  /PRINT=GOODFIT SUMMARY CI(95)
  /CRITERIA=PIN(0.05) POUT(0.10) ITERATE(20) CUT(0.5).
```

Logistic Regression

Case Processing Summary

Unweighted Cases[a]		N	Percent
Selected Cases	Included in Analysis	2554	99.8
	Missing Cases	5	.2
	Total	2559	100.0
Unselected Cases		0	.0
Total		2559	100.0

a. If weight is in effect, see classification table for the total number of cases.

Dependent Variable Encoding

Original Value	Internal Value
Other Modern	0
Use LARC	1

Categorical Variables Codings

		Frequency	Parameter coding (1)	(2)	(3)
Region recoded	N Eastern	7	.000	.000	.000
	Coast	270	1.000	.000	.000
	Eastern	437	.000	1.000	.000
	Central	235	.000	.000	1.000
	Rift Valley	780	.000	.000	.000
	Western	257	.000	.000	.000
	Nyanza	449	.000	.000	.000
	Nairobi	119	.000	.000	.000
Religion	None/other	33	.000	.000	.000
	Catholic	539	1.000	.000	.000
	Protestant/other christian	1845	.000	1.000	.000
	Muslim	137	.000	.000	1.000
Wealth status	Lower	952	.000	.000	
	Middle	566	1.000	.000	
	Higher	1036	.000	1.000	
Living children	No children	442	.000	.000	
	1-2 children	1815	1.000	.000	
	3+children	297	.000	1.000	
Marital status	Mar/ living together	1706	1.000		
	Not married/Not Living Together	848	.000		
Type of place of residence	Urban	1128	.000		
	Rural	1426	1.000		
Education recoded	None/Pri	1398	.000		
	Sec/High	1156	1.000		
Desire for children	Wants	972	1.000		
	Do not want	1582	.000		
Age in 5-year groups	15-19	517	1.000		
	20-24	2037	.000		

Categorical Variables Codings

		Parameter coding			
		(4)	(5)	(6)	(7)
Region recoded	N Eastern	.000	.000	.000	.000
	Coast	.000	.000	.000	.000
	Eastern	.000	.000	.000	.000
	Central	.000	.000	.000	.000
	Rift Valley	1.000	.000	.000	.000
	Western	.000	1.000	.000	.000
	Nyanza	.000	.000	1.000	.000
	Nairobi	.000	.000	.000	1.000
Religion	None/other				
	Catholic				
	Protestant/other christian				
	Muslim				
Wealth status	Lower				
	Middle				
	Higher				
Living children	No children				
	1-2 children				
	3+children				
Marital status	Mar/ living together				
	Not married/Not Living Together				
Type of place of residence	Urban				
	Rural				
Education recoded	None/Pri				
	Sec/High				
Desire for children	Wants				
	Do not want				
Age in 5-year groups	15-19				
	20-24				

Block 0: Beginning Block

Classification Table[a,b]

Observed			Predicted: Use LARC: Other Modern	Predicted: Use LARC: Use LARC	Percentage Correct
Step 0	Use LARC	Other Modern	2441	0	100.0
		Use LARC	536	0	.0
	Overall Percentage				82.0

a. Constant is included in the model.

b. The cut value is .500

Variables in the Equation

		B	S.E.	Wald	df	Sig.	Exp(B)
Step 0	Constant	-1.517	.048	1010.594	1	.000	.219

Variables not in the Equation

			Score	df	Sig.
Step 0	Variables	V013(1)	14.769	1	.000
		V025(1)	8.633	1	.003
		v106r(1)	5.036	1	.025
		v190r	.961	2	.618
		v190r(1)	.320	1	.572
		v190r(2)	.163	1	.687
		v218r	138.209	2	.000
		v218r(1)	48.474	1	.000
		v218r(2)	22.184	1	.000
		v501r(1)	10.855	1	.001
		v605r(1)	9.619	1	.002
		v024r	96.860	7	.000
		v024r(1)	19.853	1	.000
		v024r(2)	13.369	1	.000
		v024r(3)	.665	1	.415
		v024r(4)	44.330	1	.000
		v024r(5)	19.549	1	.000
		v024r(6)	18.229	1	.000
		v024r(7)	.050	1	.823
		v130r	21.441	3	.000
		v130r(1)	.073	1	.787
		v130r(2)	5.685	1	.017
		v130r(3)	15.705	1	.000
	Overall Statistics		275.047	19	.000

Block 1: Method = Enter

Omnibus Tests of Model Coefficients

		Chi-square	df	Sig.
Step 1	Step	330.229	19	.000
	Block	330.229	19	.000
	Model	330.229	19	.000

Model Summary

Step	-2 Log likelihood	Cox & Snell R Square	Nagelkerke R Square
1	2475.725[a]	.105	.172

a. Estimation terminated at iteration number 7 because parameter estimates changed by less than .001.

Hosmer and Lemeshow Test

Step	Chi-square	df	Sig.
1	25.004	8	.002

Contingency Table for Hosmer and Lemeshow Test

		Use LARC = Other Modern		Use LARC = Use LARC		Total
		Observed	Expected	Observed	Expected	
Step 1	1	294	295.733	5	3.442	299
	2	292	290.343	6	8.116	298
	3	270	270.266	26	25.444	296
	4	256	262.958	41	34.315	297
	5	261	251.758	35	44.405	296
	6	228	236.938	66	56.865	294
	7	227	227.910	69	68.259	296
	8	249	221.709	54	81.168	303
	9	187	205.437	112	93.430	299
	10	176	178.180	122	120.194	298

Classification Table[a]

Observed			Predicted		
			Use LARC		Percentage Correct
			Other Modern	Use LARC	
Step 1	Use LARC	Other Modern	2431	10	99.6
		Use LARC	518	18	3.3
	Overall Percentage				82.3

a. The cut value is .500

Variables in the Equation

		B	S.E.	Wald	df	Sig.	Exp(B)	95% C.I....
								Lower
Step 1[a]	V013(1)	-.308	.149	4.273	1	.039	.735	.549
	V025(1)	-.395	.127	9.635	1	.002	.674	.525
	v106r(1)	.098	.110	.794	1	.373	1.103	.889
	v190r			1.649	2	.438		
	v190r(1)	.136	.147	.860	1	.354	1.146	.859
	v190r(2)	.182	.147	1.535	1	.215	1.199	.900
	v218r			85.561	2	.000		
	v218r(1)	2.869	.316	82.320	1	.000	17.624	9.482
	v218r(2)	3.158	.354	79.479	1	.000	23.531	11.751
	v501r(1)	-.293	.118	6.164	1	.013	.746	.592
	v605r(1)	-.164	.109	2.283	1	.131	.849	.686
	v024r			83.745	7	.000		
	v024r(1)	-.500	1.678	.089	1	.766	.606	.023
	v024r(2)	-1.294	1.679	.594	1	.441	.274	.010
	v024r(3)	-.714	1.679	.181	1	.670	.490	.018
	v024r(4)	-1.553	1.677	.858	1	.354	.212	.008
	v024r(5)	-.254	1.679	.023	1	.880	.775	.029
	v024r(6)	-.357	1.676	.045	1	.831	.700	.026
	v024r(7)	-1.055	1.678	.395	1	.530	.348	.013
	v130r			12.729	3	.005		
	v130r(1)	-.776	.422	3.392	1	.066	.460	.201
	v130r(2)	-.977	.411	5.656	1	.017	.377	.168
	v130r(3)	-.290	.458	.400	1	.527	.748	.305
	Constant	-2.030	1.753	1.341	1	.247	.131	

Variables in the Equation

		95% C.I....
		Upper
Step 1[a]	V013(1)	.984
	V025(1)	.865
	v106r(1)	1.367
	v190r	
	v190r(1)	1.528
	v190r(2)	1.598
	v218r	
	v218r(1)	32.756
	v218r(2)	47.119
	v501r(1)	.940
	v605r(1)	1.050
	v024r	
	v024r(1)	16.262
	v024r(2)	7.363
	v024r(3)	13.143
	v024r(4)	5.662
	v024r(5)	20.813
	v024r(6)	18.702
	v024r(7)	9.333
	v130r	
	v130r(1)	1.051
	v130r(2)	.842
	v130r(3)	1.837
	Constant	

a. Variable(s) entered on step 1: V013, V025, v106r, v190r, v218r, v501r, v605r, v024r, v130r.

```
LOGISTIC REGRESSION VARIABLES v312r
 /METHOD=ENTER V013 V025 v106r v190r v218r v501r v605r v024r
 /CONTRAST (V013)=Indicator
 /CONTRAST (V025)=Indicator(1)
 /CONTRAST (v106r)=Indicator(1)
 /CONTRAST (v190r)=Indicator(1)
 /CONTRAST (v218r)=Indicator(1)
 /CONTRAST (v501r)=Indicator
 /CONTRAST (v605r)=Indicator
 /CONTRAST (v024r)=Indicator(1)
 /PRINT=GOODFIT SUMMARY CI(95)
 /CRITERIA=PIN(0.05) POUT(0.10) ITERATE(20) CUT(0.5).
```

Logistic Regression

Case Processing Summary

Unweighted Cases[a]		N	Percent
Selected Cases	Included in Analysis	2559	100.0
	Missing Cases	0	.0
	Total	2559	100.0
Unselected Cases		0	.0
Total		2559	100.0

a. If weight is in effect, see classification table for the total number of cases.

Dependent Variable Encoding

Original Value	Internal Value
Other Modern	0
Use LARC	1

Categorical Variables Codings

		Frequency	Parameter coding		
			(1)	(2)	(3)
Region recoded	N Eastern	7	.000	.000	.000
	Coast	270	1.000	.000	.000
	Eastern	438	.000	1.000	.000
	Central	235	.000	.000	1.000
	Rift Valley	781	.000	.000	.000
	Western	258	.000	.000	.000
	Nyanza	451	.000	.000	.000
	Nairobi	119	.000	.000	.000
Wealth status	Lower	955	.000	.000	
	Middle	566	1.000	.000	
	Higher	1038	.000	1.000	
Living children	No children	443	.000	.000	
	1-2 children	1819	1.000	.000	
	3+children	297	.000	1.000	
Marital status	Mar/ living together	1710	1.000		
	Not married/Not Living Together	849	.000		
Type of place of residence	Urban	1128	.000		
	Rural	1431	1.000		
Education recoded	None/Pri	1401	.000		
	Sec/High	1158	1.000		

Categorical Variables Codings

		Parameter coding			
		(4)	(5)	(6)	(7)
Region recoded	N Eastern	.000	.000	.000	.000
	Coast	.000	.000	.000	.000
	Eastern	.000	.000	.000	.000
	Central	.000	.000	.000	.000
	Rift Valley	1.000	.000	.000	.000
	Western	.000	1.000	.000	.000
	Nyanza	.000	.000	1.000	.000
	Nairobi	.000	.000	.000	1.000
Wealth status	Lower				
	Middle				
	Higher				
Living children	No children				
	1-2 children				
	3+children				
Marital status	Mar/ living together				
	Not married/Not Living Together				
Type of place of residence	Urban				
	Rural				
Education recoded	None/Pri				
	Sec/High				

Categorical Variables Codings

			Parameter coding		
		Frequency	(1)	(2)	(3)
Desire for children	Wants	972	1.000		
	Do not want	1587	.000		
Age in 5-year groups	15-19	518	1.000		
	20-24	2041	.000		

Categorical Variables Codings

		Parameter coding			
		(4)	(5)	(6)	(7)
Desire for children	Wants				
	Do not want				
Age in 5-year groups	15-19				
	20-24				

Block 0: Beginning Block

Classification Table[a,b]

Observed			Predicted: Use LARC: Other Modern	Predicted: Use LARC: Use LARC	Predicted: Percentage Correct
Step 0	Use LARC	Other Modern	2445	0	100.0
		Use LARC	537	0	.0
	Overall Percentage				82.0

a. Constant is included in the model.

b. The cut value is .500

Variables in the Equation

		B	S.E.	Wald	df	Sig.	Exp(B)
Step 0	Constant	-1.516	.048	1011.767	1	.000	.220

Variables not in the Equation

			Score	df	Sig.
Step 0	Variables	V013(1)	14.977	1	.000
		V025(1)	8.525	1	.004
		v106r(1)	5.067	1	.024
		v190r	.925	2	.630
		v190r(1)	.310	1	.578
		v190r(2)	.155	1	.694
		v218r	138.653	2	.000
		v218r(1)	48.868	1	.000
		v218r(2)	22.121	1	.000
		v501r(1)	11.070	1	.001
		v605r(1)	9.682	1	.002
		v024r	98.015	7	.000
		v024r(1)	19.797	1	.000
		v024r(2)	13.572	1	.000
		v024r(3)	.655	1	.418
		v024r(4)	44.708	1	.000
		v024r(5)	20.448	1	.000
		v024r(6)	18.179	1	.000
		v024r(7)	.053	1	.818
	Overall Statistics		263.622	16	.000

Block 1: Method = Enter

Omnibus Tests of Model Coefficients

		Chi-square	df	Sig.
Step 1	Step	319.526	16	.000
	Block	319.526	16	.000
	Model	319.526	16	.000

Model Summary

Step	-2 Log likelihood	Cox & Snell R Square	Nagelkerke R Square
1	2492.164[a]	.102	.166

a. Estimation terminated at iteration number 7 because parameter estimates changed by less than .001.

Hosmer and Lemeshow Test

Step	Chi-square	df	Sig.
1	17.467	8	.026

Contingency Table for Hosmer and Lemeshow Test

		Use LARC = Other Modern		Use LARC = Use LARC		Total
		Observed	Expected	Observed	Expected	
Step 1	1	297	297.427	4	3.516	301
	2	294	292.753	7	8.299	301
	3	261	266.481	32	26.602	293
	4	266	262.021	31	34.746	297
	5	259	254.813	41	45.636	300
	6	221	235.942	73	57.685	294
	7	232	228.579	67	69.765	298
	8	240	218.262	58	79.614	298
	9	196	207.224	105	92.897	300
	10	180	181.498	120	118.116	300

Classification Table[a]

Observed			Predicted		
			Use LARC		Percentage Correct
			Other Modern	Use LARC	
Step 1	Use LARC	Other Modern	2445	0	100.0
		Use LARC	532	5	.9
	Overall Percentage				82.1

a. The cut value is .500

Variables in the Equation

		B	S.E.	Wald	df	Sig.	Exp(B)	95% C.I..
								Lower
Step 1[a]	V013(1)	-.264	.147	3.214	1	.073	.768	.576
	V025(1)	-.407	.127	10.280	1	.001	.666	.519
	v106r(1)	.083	.109	.576	1	.448	1.086	.877
	v190r			1.345	2	.511		
	v190r(1)	.123	.146	.717	1	.397	1.131	.850
	v190r(2)	.162	.145	1.240	1	.265	1.176	.884
	v218r			85.411	2	.000		
	v218r(1)	2.864	.316	82.150	1	.000	17.525	9.435
	v218r(2)	3.151	.354	79.414	1	.000	23.349	11.677
	v501r(1)	-.281	.117	5.733	1	.017	.755	.600
	v605r(1)	-.158	.108	2.130	1	.144	.854	.691
	v024r			90.335	7	.000		
	v024r(1)	-.410	1.688	.059	1	.808	.664	.024
	v024r(2)	-1.429	1.688	.716	1	.397	.240	.009
	v024r(3)	-.854	1.688	.256	1	.613	.426	.016
	v024r(4)	-1.671	1.686	.982	1	.322	.188	.007
	v024r(5)	-.402	1.688	.057	1	.812	.669	.024
	v024r(6)	-.503	1.686	.089	1	.765	.604	.022
	v024r(7)	-1.175	1.687	.486	1	.486	.309	.011
	Constant	-2.794	1.714	2.658	1	.103	.061	

Variables in the Equation

		95% C.I....
		Upper
Step 1[a]	V013(1)	1.025
	V025(1)	.854
	v106r(1)	1.344
	v190r	
	v190r(1)	1.506
	v190r(2)	1.564
	v218r	
	v218r(1)	32.554
	v218r(2)	46.687
	v501r(1)	.950
	v605r(1)	1.056
	v024r	
	v024r(1)	18.140
	v024r(2)	6.555
	v024r(3)	11.634
	v024r(4)	5.125
	v024r(5)	18.289
	v024r(6)	16.448
	v024r(7)	8.423
	Constant	

a. Variable(s) entered on step 1: V013, V025, v106r, v190r, v218r, v501r, v605r, v024r.

```
CROSSTABS
 /TABLES=v312r BY v024r
 /FORMAT=AVALUE TABLES
 /STATISTICS=CHISQ
 /CELLS=COUNT ROW
 /COUNT ROUND CELL.
```

Crosstabs

Case Processing Summary

	Cases					
	Valid		Missing		Total	
	N	Percent	N	Percent	N	Percent
Use LARC * Region recoded	2981	100.0%	.873	0.0%	2981.873	100.0%

Use LARC * Region recoded Crosstabulation

			Region recoded			
			N Eastern	Coast	Eastern	Central
Use LARC	Other Modern	Count	1	176	396	275
		% within Use LARC	0.0%	7.2%	16.2%	11.2%
	Use LARC	Count	1	70	53	67
		% within Use LARC	0.2%	13.1%	9.9%	12.5%
Total		Count	2	246	449	342
		% within Use LARC	0.1%	8.3%	15.1%	11.5%

Use LARC * Region recoded Crosstabulation

			Region recoded			
			Rift Valley	Western	Nyanza	Nairobi
Use LARC	Other Modern	Count	699	209	320	369
		% within Use LARC	28.6%	8.5%	13.1%	15.1%
	Use LARC	Count	78	80	108	79
		% within Use LARC	14.6%	14.9%	20.1%	14.7%
Total		Count	777	289	428	448
		% within Use LARC	26.1%	9.7%	14.4%	15.0%

Use LARC * Region recoded Crosstabulation

			Total
Use LARC	Other Modern	Count	2445
		% within Use LARC	100.0%
	Use LARC	Count	536
		% within Use LARC	100.0%
Total		Count	2981
		% within Use LARC	100.0%

Chi-Square Tests

	Value	df	Asymp. Sig. (2-sided)
Pearson Chi-Square	98.887[a]	7	.000
Likelihood Ratio	99.391	7	.000
Linear-by-Linear Association	2.025	1	.155
N of Valid Cases	2981		

a. 2 cells (12.5%) have expected count less than 5. The minimum expected count is .36.

```
LOGISTIC REGRESSION VARIABLES v312r
 /METHOD=ENTER V013 V025 v106r v190r v218r v501r v605r v024r
 /CONTRAST (V013)=Indicator
 /CONTRAST (V025)=Indicator(1)
 /CONTRAST (v106r)=Indicator(1)
 /CONTRAST (v190r)=Indicator(1)
 /CONTRAST (v218r)=Indicator(1)
 /CONTRAST (v501r)=Indicator
 /CONTRAST (v605r)=Indicator
 /CONTRAST (v024r)=Indicator(1)
 /PRINT=GOODFIT SUMMARY CI(95)
 /CRITERIA=PIN(0.05) POUT(0.10) ITERATE(20) CUT(0.5).
```

Logistic Regression

Case Processing Summary

Unweighted Cases[a]		N	Percent
Selected Cases	Included in Analysis	2559	100.0
	Missing Cases	0	.0
	Total	2559	100.0
Unselected Cases		0	.0
Total		2559	100.0

a. If weight is in effect, see classification table for the total number of cases.

Dependent Variable Encoding

Original Value	Internal Value
Other Modern	0
Use LARC	1

Categorical Variables Codings

		Frequency	Parameter coding		
			(1)	(2)	(3)
Region recoded	N Eastern	7	.000	.000	.000
	Coast	270	1.000	.000	.000
	Eastern	438	.000	1.000	.000
	Central	235	.000	.000	1.000
	Rift Valley	781	.000	.000	.000
	Western	258	.000	.000	.000
	Nyanza	451	.000	.000	.000
	Nairobi	119	.000	.000	.000
Wealth status	Lower	955	.000	.000	
	Middle	566	1.000	.000	
	Higher	1038	.000	1.000	
Living children	No children	443	.000	.000	
	1-2 children	1819	1.000	.000	
	3+children	297	.000	1.000	
Marital status	Mar/ living together	1710	1.000		
	Not married/Not Living Together	849	.000		
Type of place of residence	Urban	1128	.000		
	Rural	1431	1.000		
Education recoded	None/Pri	1401	.000		
	Sec/High	1158	1.000		
Desire for children	Wants	972	1.000		
	Do not want	1587	.000		
Age in 5-year groups	15-19	518	1.000		
	20-24	2041	.000		

Categorical Variables Codings

		Parameter coding			
		(4)	(5)	(6)	(7)
Region recoded	N Eastern	.000	.000	.000	.000
	Coast	.000	.000	.000	.000
	Eastern	.000	.000	.000	.000
	Central	.000	.000	.000	.000
	Rift Valley	1.000	.000	.000	.000
	Western	.000	1.000	.000	.000
	Nyanza	.000	.000	1.000	.000
	Nairobi	.000	.000	.000	1.000
Wealth status	Lower				
	Middle				
	Higher				
Living children	No children				
	1-2 children				
	3+children				
Marital status	Mar/ living together				
	Not married/Not Living Together				
Type of place of residence	Urban				
	Rural				
Education recoded	None/Pri				
	Sec/High				
Desire for children	Wants				
	Do not want				
Age in 5-year groups	15-19				
	20-24				

Block 0: Beginning Block

Classification Table[a,b]

Observed			Predicted		
			Use LARC		Percentage Correct
			Other Modern	Use LARC	
Step 0	Use LARC	Other Modern	2445	0	100.0
		Use LARC	537	0	.0
	Overall Percentage				82.0

a. Constant is included in the model.

b. The cut value is .500

Variables in the Equation

		B	S.E.	Wald	df	Sig.	Exp(B)
Step 0	Constant	-1.516	.048	1011.767	1	.000	.220

Variables not in the Equation

			Score	df	Sig.
Step 0	Variables	V013(1)	14.977	1	.000
		V025(1)	8.525	1	.004
		v106r(1)	5.067	1	.024
		v190r	.925	2	.630
		v190r(1)	.310	1	.578
		v190r(2)	.155	1	.694
		v218r	138.653	2	.000
		v218r(1)	48.868	1	.000
		v218r(2)	22.121	1	.000
		v501r(1)	11.070	1	.001
		v605r(1)	9.682	1	.002
		v024r	98.015	7	.000
		v024r(1)	19.797	1	.000
		v024r(2)	13.572	1	.000
		v024r(3)	.655	1	.418
		v024r(4)	44.708	1	.000
		v024r(5)	20.448	1	.000
		v024r(6)	18.179	1	.000
		v024r(7)	.053	1	.818
	Overall Statistics		263.622	16	.000

Block 1: Method = Enter

Omnibus Tests of Model Coefficients

		Chi-square	df	Sig.
Step 1	Step	319.526	16	.000
	Block	319.526	16	.000
	Model	319.526	16	.000

Model Summary

Step	-2 Log likelihood	Cox & Snell R Square	Nagelkerke R Square
1	2492.164[a]	.102	.166

a. Estimation terminated at iteration number 7 because parameter estimates changed by less than .001.

Hosmer and Lemeshow Test

Step	Chi-square	df	Sig.
1	17.467	8	.026

Contingency Table for Hosmer and Lemeshow Test

		Use LARC = Other Modern		Use LARC = Use LARC		
		Observed	Expected	Observed	Expected	Total
Step 1	1	297	297.427	4	3.516	301
	2	294	292.753	7	8.299	301
	3	261	266.481	32	26.602	293
	4	266	262.021	31	34.746	297
	5	259	254.813	41	45.636	300
	6	221	235.942	73	57.685	294
	7	232	228.579	67	69.765	298
	8	240	218.262	58	79.614	298
	9	196	207.224	105	92.897	300
	10	180	181.498	120	118.116	300

Classification Table[a]

			Predicted		
			Use LARC		Percentage Correct
Observed			Other Modern	Use LARC	
Step 1	Use LARC	Other Modern	2445	0	100.0
		Use LARC	532	5	.9
	Overall Percentage				82.1

a. The cut value is .500

Variables in the Equation

		B	S.E.	Wald	df	Sig.	Exp(B)	95% C.I..
								Lower
Step 1[a]	V013(1)	-.264	.147	3.214	1	.073	.768	.576
	V025(1)	-.407	.127	10.280	1	.001	.666	.519
	v106r(1)	.083	.109	.576	1	.448	1.086	.877
	v190r			1.345	2	.511		
	v190r(1)	.123	.146	.717	1	.397	1.131	.850
	v190r(2)	.162	.145	1.240	1	.265	1.176	.884
	v218r			85.411	2	.000		
	v218r(1)	2.864	.316	82.150	1	.000	17.525	9.435
	v218r(2)	3.151	.354	79.414	1	.000	23.349	11.677
	v501r(1)	-.281	.117	5.733	1	.017	.755	.600
	v605r(1)	-.158	.108	2.130	1	.144	.854	.691
	v024r			90.335	7	.000		
	v024r(1)	-.410	1.688	.059	1	.808	.664	.024
	v024r(2)	-1.429	1.688	.716	1	.397	.240	.009
	v024r(3)	-.854	1.688	.256	1	.613	.426	.016
	v024r(4)	-1.671	1.686	.982	1	.322	.188	.007
	v024r(5)	-.402	1.688	.057	1	.812	.669	.024
	v024r(6)	-.503	1.686	.089	1	.765	.604	.022
	v024r(7)	-1.175	1.687	.486	1	.486	.309	.011
	Constant	-2.794	1.714	2.658	1	.103	.061	

Variables in the Equation

		95% C.I....
		Upper
Step 1[a]	V013(1)	1.025
	V025(1)	.854
	v106r(1)	1.344
	v190r	
	v190r(1)	1.506
	v190r(2)	1.564
	v218r	
	v218r(1)	32.554
	v218r(2)	46.687
	v501r(1)	.950
	v605r(1)	1.056
	v024r	
	v024r(1)	18.140
	v024r(2)	6.555
	v024r(3)	11.634
	v024r(4)	5.125
	v024r(5)	18.289
	v024r(6)	16.448
	v024r(7)	8.423
	Constant	

a. Variable(s) entered on step 1: V013, V025, v106r, v190r, v218r, v501r, v605r, v024r.

```
FREQUENCIES VARIABLE = v106.
```

Frequencies

Statistics

Highest educational level

N	Valid	2982
	Missing	0

Highest educational level

		Frequency	Percent	Valid Percent	Cumulative Percent
Valid	No education	47	1.6	1.6	1.6
	Primary	1441	48.3	48.3	49.9
	Secondary	1192	40.0	40.0	89.9
	Higher	302	10.1	10.1	100.0
	Total	2982	100.0	100.0	

```
 /ORDER=ANALYSIS.

>Error # 1.  Command name: /ORDER
>The first word in the line is not recognized as an SPSS Statistics command.
>Execution of this command stops.
```

```
 CROSSTABS
  /TABLES=v312r BY v024r V013 V025 v190r v218r v130r v501r v605r v106r
  /FORMAT=AVALUE TABLES
  /STATISTICS=CHISQ
  /CELLS=COUNT ROW COLUMN
  /COUNT ROUND CELL.
```

Crosstabs

Case Processing Summary

	Cases					
	Valid		Missing		Total	
	N	Percent	N	Percent	N	Percent
Use LARC * Region recoded	2981	100.0%	.873	0.0%	2981.873	100.0%
Use LARC * Age in 5-year groups	2981	100.0%	.873	0.0%	2981.873	100.0%
Use LARC * Type of place of residence	2982[a]	100.0%	0	0.0%	2981.873	100.0%
Use LARC * Wealth status	2981[a]	100.0%	.873	0.0%	2981.873	100.0%
Use LARC * Living children	2982[a]	100.0%	0	0.0%	2981.873	100.0%
Use LARC * Religion	2977[a]	99.8%	4.873	0.2%	2981.873	100.0%
Use LARC * Marital status	2981[a]	100.0%	.873	0.0%	2981.873	100.0%
Use LARC * Desire for children	2981[a]	100.0%	.873	0.0%	2981.873	100.0%
Use LARC * Education recoded	2982[a]	100.0%	0	0.0%	2981.873	100.0%

a. Number of valid cases is different from the total count in the crosstabulation table because the cell counts have been rounded.

Use LARC * Region recoded

Crosstab

			Region recoded			
			N Eastern	Coast	Eastern	Central
Use LARC	Other Modern	Count	1	176	396	275
		% within Use LARC	0.0%	7.2%	16.2%	11.2%
		% within Region recoded	50.0%	71.5%	88.2%	80.4%
	Use LARC	Count	1	70	53	67
		% within Use LARC	0.2%	13.1%	9.9%	12.5%
		% within Region recoded	50.0%	28.5%	11.8%	19.6%
Total		Count	2	246	449	342
		% within Use LARC	0.1%	8.3%	15.1%	11.5%
		% within Region recoded	100.0%	100.0%	100.0%	100.0%

Crosstab

			Region recoded			
			Rift Valley	Western	Nyanza	Nairobi
Use LARC	Other Modern	Count	699	209	320	369
		% within Use LARC	28.6%	8.5%	13.1%	15.1%
		% within Region recoded	90.0%	72.3%	74.8%	82.4%
	Use LARC	Count	78	80	108	79
		% within Use LARC	14.6%	14.9%	20.1%	14.7%
		% within Region recoded	10.0%	27.7%	25.2%	17.6%
Total		Count	777	289	428	448
		% within Use LARC	26.1%	9.7%	14.4%	15.0%
		% within Region recoded	100.0%	100.0%	100.0%	100.0%

Crosstab

			Total
Use LARC	Other Modern	Count	2445
		% within Use LARC	100.0%
		% within Region recoded	82.0%
	Use LARC	Count	536
		% within Use LARC	100.0%
		% within Region recoded	18.0%
Total		Count	2981
		% within Use LARC	100.0%
		% within Region recoded	100.0%

Chi-Square Tests

	Value	df	Asymp. Sig. (2-sided)
Pearson Chi-Square	98.887[a]	7	.000
Likelihood Ratio	99.391	7	.000
Linear-by-Linear Association	2.025	1	.155
N of Valid Cases	2981		

a. 2 cells (12.5%) have expected count less than 5. The minimum expected count is .36.

Use LARC * Age in 5-year groups

Crosstab

			Age in 5-year groups		Total
			15-19	20-24	
Use LARC	Other Modern	Count	513	1932	2445
		% within Use LARC	21.0%	79.0%	100.0%
		% within Age in 5-year groups	87.5%	80.7%	82.0%
	Use LARC	Count	73	463	536
		% within Use LARC	13.6%	86.4%	100.0%
		% within Age in 5-year groups	12.5%	19.3%	18.0%
Total		Count	586	2395	2981
		% within Use LARC	19.7%	80.3%	100.0%
		% within Age in 5-year groups	100.0%	100.0%	100.0%

Chi-Square Tests

	Value	df	Asymp. Sig. (2-sided)	Exact Sig. (2-sided)	Exact Sig. (1-sided)
Pearson Chi-Square	15.087[a]	1	.000		
Continuity Correction[b]	14.625	1	.000		
Likelihood Ratio	16.195	1	.000		
Fisher's Exact Test				.000	.000
Linear-by-Linear Association	15.082	1	.000		
N of Valid Cases	2981				

a. 0 cells (.0%) have expected count less than 5. The minimum expected count is 105.37.

b. Computed only for a 2x2 table

Use LARC * Type of place of residence

Crosstab

			Type of place of residence		Total
			Urban	Rural	
Use LARC	Other Modern	Count	1152	1293	2445
		% within Use LARC	47.1%	52.9%	100.0%
		% within Type of place of residence	79.9%	84.0%	82.0%
	Use LARC	Count	290	247	537
		% within Use LARC	54.0%	46.0%	100.0%
		% within Type of place of residence	20.1%	16.0%	18.0%
Total		Count	1442	1540	2982
		% within Use LARC	48.4%	51.6%	100.0%
		% within Type of place of residence	100.0%	100.0%	100.0%

Chi-Square Tests

	Value	df	Asymp. Sig. (2-sided)	Exact Sig. (2-sided)	Exact Sig. (1-sided)
Pearson Chi-Square	8.363[a]	1	.004		
Continuity Correction[b]	8.089	1	.004		
Likelihood Ratio	8.361	1	.004		
Fisher's Exact Test				.004	.002
Linear-by-Linear Association	8.360	1	.004		
N of Valid Cases	2982				

a. 0 cells (.0%) have expected count less than 5. The minimum expected count is 259.68.

b. Computed only for a 2x2 table

Use LARC * Wealth status

Crosstab

			Wealth status			Total
			Lower	Middle	Higher	
Use LARC	Other Modern	Count	739	508	1198	2445
		% within Use LARC	30.2%	20.8%	49.0%	100.0%
		% within Wealth status	83.0%	81.3%	81.7%	82.0%
	Use LARC	Count	151	117	268	536
		% within Use LARC	28.2%	21.8%	50.0%	100.0%
		% within Wealth status	17.0%	18.7%	18.3%	18.0%
Total		Count	890	625	1466	2981
		% within Use LARC	29.9%	21.0%	49.2%	100.0%
		% within Wealth status	100.0%	100.0%	100.0%	100.0%

Chi-Square Tests

	Value	df	Asymp. Sig. (2-sided)
Pearson Chi-Square	.942[a]	2	.624
Likelihood Ratio	.949	2	.622
Linear-by-Linear Association	.545	1	.460
N of Valid Cases	2981		

a. 0 cells (.0%) have expected count less than 5. The minimum expected count is 112.38.

Use LARC * Living children

Crosstab

			Living children		
			No children	1-2 children	3+children
Use LARC	Other Modern	Count	583	1661	202
		% within Use LARC	23.8%	67.9%	8.3%
		% within Living children	98.1%	78.8%	71.9%
	Use LARC	Count	11	446	79
		% within Use LARC	2.1%	83.2%	14.7%
		% within Living children	1.9%	21.2%	28.1%
Total		Count	594	2107	281
		% within Use LARC	19.9%	70.7%	9.4%
		% within Living children	100.0%	100.0%	100.0%

Crosstab

			Total
Use LARC	Other Modern	Count	2446
		% within Use LARC	100.0%
		% within Living children	82.0%
	Use LARC	Count	536
		% within Use LARC	100.0%
		% within Living children	18.0%
Total		Count	2982
		% within Use LARC	100.0%
		% within Living children	100.0%

Chi-Square Tests

	Value	df	Asymp. Sig. (2-sided)
Pearson Chi-Square	138.890[a]	2	.000
Likelihood Ratio	190.558	2	.000
Linear-by-Linear Association	124.315	1	.000
N of Valid Cases	2982		

a. 0 cells (.0%) have expected count less than 5. The minimum expected count is 50.51.

Use LARC * Religion

Crosstab

			Religion			
			None/other	Catholic	Protestant/other christian	Muslim
Use LARC	Other Modern	Count	22	538	1814	67
		% within Use LARC	0.9%	22.0%	74.3%	2.7%
		% within Religion	66.7%	81.6%	83.0%	67.0%
	Use LARC	Count	11	121	371	33
		% within Use LARC	2.1%	22.6%	69.2%	6.2%
		% within Religion	33.3%	18.4%	17.0%	33.0%
Total		Count	33	659	2185	100
		% within Use LARC	1.1%	22.1%	73.4%	3.4%
		% within Religion	100.0%	100.0%	100.0%	100.0%

Crosstab

			Total
Use LARC	Other Modern	Count	2441
		% within Use LARC	100.0%
		% within Religion	82.0%
	Use LARC	Count	536
		% within Use LARC	100.0%
		% within Religion	18.0%
Total		Count	2977
		% within Use LARC	100.0%
		% within Religion	100.0%

Chi-Square Tests

	Value	df	Asymp. Sig. (2-sided)
Pearson Chi-Square	22.096[a]	3	.000
Likelihood Ratio	19.004	3	.000
Linear-by-Linear Association	.057	1	.811
N of Valid Cases	2977		

a. 0 cells (.0%) have expected count less than 5. The minimum expected count is 5.94.

Use LARC * Marital status

Crosstab

			Marital status		Total
			Mar/ living together	Not married/Not Living Together	
Use LARC	Other Modern	Count	1562	883	2445
		% within Use LARC	63.9%	36.1%	100.0%
		% within Marital status	80.3%	85.2%	82.0%
	Use LARC	Count	383	153	536
		% within Use LARC	71.5%	28.5%	100.0%
		% within Marital status	19.7%	14.8%	18.0%
Total		Count	1945	1036	2981
		% within Use LARC	65.2%	34.8%	100.0%
		% within Marital status	100.0%	100.0%	100.0%

Chi-Square Tests

	Value	df	Asymp. Sig. (2-sided)	Exact Sig. (2-sided)	Exact Sig. (1-sided)
Pearson Chi-Square	11.109[a]	1	.001		
Continuity Correction[b]	10.778	1	.001		
Likelihood Ratio	11.391	1	.001		
Fisher's Exact Test				.001	.000
Linear-by-Linear Association	11.106	1	.001		
N of Valid Cases	2981				

a. 0 cells (.0%) have expected count less than 5. The minimum expected count is 186.28.

b. Computed only for a 2x2 table

Use LARC * Desire for children

Crosstab

			Desire for children		Total
			Wants	Do not want	
Use LARC	Other Modern	Count	956	1489	2445
		% within Use LARC	39.1%	60.9%	100.0%
		% within Desire for children	84.8%	80.3%	82.0%
	Use LARC	Count	171	365	536
		% within Use LARC	31.9%	68.1%	100.0%
		% within Desire for children	15.2%	19.7%	18.0%
Total		Count	1127	1854	2981
		% within Use LARC	37.8%	62.2%	100.0%
		% within Desire for children	100.0%	100.0%	100.0%

Chi-Square Tests

	Value	df	Asymp. Sig. (2-sided)	Exact Sig. (2-sided)	Exact Sig. (1-sided)
Pearson Chi-Square	9.685[a]	1	.002		
Continuity Correction[b]	9.381	1	.002		
Likelihood Ratio	9.866	1	.002		
Fisher's Exact Test				.002	.001
Linear-by-Linear Association	9.682	1	.002		
N of Valid Cases	2981				

a. 0 cells (.0%) have expected count less than 5. The minimum expected count is 202.64.

b. Computed only for a 2x2 table

Use LARC * Education recoded

Crosstab

			Education recoded		Total
			None/Pri	Sec/High	
Use LARC	Other Modern	Count	1196	1249	2445
		% within Use LARC	48.9%	51.1%	100.0%
		% within Education recoded	80.4%	83.6%	82.0%
	Use LARC	Count	292	245	537
		% within Use LARC	54.4%	45.6%	100.0%
		% within Education recoded	19.6%	16.4%	18.0%
Total		Count	1488	1494	2982
		% within Use LARC	49.9%	50.1%	100.0%
		% within Education recoded	100.0%	100.0%	100.0%

Chi-Square Tests

	Value	df	Asymp. Sig. (2-sided)	Exact Sig. (2-sided)	Exact Sig. (1-sided)
Pearson Chi-Square	5.250[a]	1	.022		
Continuity Correction[b]	5.034	1	.025		
Likelihood Ratio	5.256	1	.022		
Fisher's Exact Test				.022	.012
Linear-by-Linear Association	5.249	1	.022		
N of Valid Cases	2982				

a. 0 cells (.0%) have expected count less than 5. The minimum expected count is 267.96.

b. Computed only for a 2x2 table

```
 RECODE v024 (3=1) (4=1) ( 9=1) (1=2) (2=2) (5=2) (7=2) (8=2) into v024r.
VARIABLE LABELS v024r "Region recoded".
VALUE LABELS v024r 1 "High Contraception" 2 "Low Contraception" .
CROSSTABS
  /TABLES=v312r BY v024r
  /FORMAT=AVALUE TABLES
  /STATISTICS=CHISQ
  /CELLS=COUNT ROW COLUMN
  /COUNT ROUND CELL.
```

Crosstabs

Case Processing Summary

	Cases					
	Valid		Missing		Total	
	N	Percent	N	Percent	N	Percent
Use LARC * Region recoded	2982	100.0%	0	0.0%	2981.873	100.0%

Use LARC * Region recoded Crosstabulation

			Region recoded		
			High Contraception	Low Contraception	Total
Use LARC	Other Modern	Count	1040	1405	2445
		% within Use LARC	42.5%	57.5%	100.0%
		% within Region recoded	83.9%	80.6%	82.0%
	Use LARC	Count	199	338	537
		% within Use LARC	37.1%	62.9%	100.0%
		% within Region recoded	16.1%	19.4%	18.0%
Total		Count	1239	1743	2982
		% within Use LARC	41.5%	58.5%	100.0%
		% within Region recoded	100.0%	100.0%	100.0%

Chi-Square Tests

	Value	df	Asymp. Sig. (2-sided)	Exact Sig. (2-sided)	Exact Sig. (1-sided)
Pearson Chi-Square	5.441[a]	1	.020		
Continuity Correction[b]	5.217	1	.022		
Likelihood Ratio	5.492	1	.019		
Fisher's Exact Test				.020	.011
Linear-by-Linear Association	5.439	1	.020		
N of Valid Cases	2982				

a. 0 cells (.0%) have expected count less than 5. The minimum expected count is 223.12.

b. Computed only for a 2x2 table
